# Supplementary material for: Neuroprosthetic contact lens enabled sensorimotor system for point-of-care monitoring and feedback of intraocular pressure
Source: Nat Commun. 2024 Jul 5;15:5635. doi: 10.1038/s41467-024-49907-5 (PMC11224243; doi:10.1038/s41467-024-49907-5)
Supplement: Supplementary file 1 — Supplementary information [file 41467_2024_49907_MOESM1_ESM.pdf]

Supplementary Information for:

Neuroprosthetic contact lens enabled sensorimotor system for point-of-care monitoring and feedback of intraocular pressure

Weijia Liu[1], Zhijian Du[1], Zhongyi Duan[1], La Li[1]*, and Guozhen Shen[1]*

[1] School of Integrated Circuits and Electronics, Beijing Institute of Technology, Beijing 100081, China. E-mail: lali@bit.edu.cn, gzshen@bit.edu.cn.

This PDF file includes:

Supplementary Methods

Supplementary Notes 1–8

Supplementary Figs. 1–41

Supplementary Table 1–6

Supplementary References 1–17

Table of Context

Supplementary Methods

Supplementary Notes:

Supplementary Note 1: Calculation.

Supplementary Note 2: Characterization of $Ti_3C_2T_x$ MXene.

Supplementary Note 3: Haze comparisons of the $Ti_3C_2T_x$-SCL devices with commercial corneal contact lenses.

Supplementary Note 4: Analog intraocular pressure testing platform.

Supplementary Note 5: Performance indicators of the $Ti_3C_2T_x$-SCL.

Supplementary Note 6: Performance testing of rectilinear $Ti_3C_2T_x$-SCL.

Supplementary Note 7: Comparison with reported articles.

Supplementary Note 8: Exploration of intraocular pressure sensorimotor circuits in the rat.

Supplementary Figures:

Supplementary Fig. 1 | The curvature changes of corneal contact lenses worn by the eye during an increase in intraocular pressure from P_1 to P_2.

Supplementary Fig. 2 | Schematic diagram of the $Ti_3C_2T_x$-SCL preparation process.

Supplementary Fig. 3 | Thickness measurement of $Ti_3C_2T_x$-SCL (left) and commercial-SCL (right).

Supplementary Fig. 4 | Average weight of $Ti_3C_2T_x$-SCL and weight comparison with commercial transparent and color contact lenses.

Supplementary Fig. 5 | Transmittance of $Ti_3C_2T_x$-SCL.

Supplementary Fig. 6 | Haze of $Ti_3C_2T_x$-SCL.

Supplementary Fig. 7 | XRD pattern.

Supplementary Fig. 8 | Raman spectra of $Ti_3C_2T_x$ MXene.

Supplementary Fig. 9 | FTIR of $Ti_3C_2T_x$ MXene.

Supplementary Fig. 10 | XPS spectra and high magnification.

Supplementary Fig. 11 | Contact angle test of $Ti_3C_2T_x$ MXene film with water.

Supplementary Fig. 12 | Simulation of eyeball deformation under different IOP.

Supplementary Fig. 13 | Finite element analysis of rectilinear and serpentine electrodes under 0%, 0.5%, 1%, 1.5% and 2% strain.

Supplementary Fig. 14 | Comparison of serpentine and rectilinear electrodes.

Supplementary Fig. 15 | Digital photograph of a simulated IOP testing platform containing bionic eyeball, motorized sliding table with program control system, manometer and source meter.

Supplementary Fig. 16 | Digital photograph of the bionic eyeball consisting of a stainless steel chamber and a intraocular pressure sensor based on full $Ti_3C_2T_x$ MXene.

Supplementary Fig. 17 | Static response curve for $Ti_3C_2T_x$-SCL.

Supplementary Fig. 18 | The smallest detection limit of the $Ti_3C_2T_x$-SCL.

Supplementary Fig. 19 | Output voltage variations and statistical distribution of the $Ti_3C_2T_x$ -SCL at 0.05, 0.1, 0.15, 0.2 and 0.25 mmHg, respectively.

Supplementary Fig. 20 | Statistical analysis of the $Ti_3C_2T_x$ -SCL accuracy at 0.2 mmHg.

Supplementary Fig. 21 | The measurement error of the $Ti_3C_2T_x$-SCL.

Supplementary Fig. 22 | Cycling test at 1x speed in the range of 0-21 mmHg.

Supplementary Fig. 23 | Performance testing of rectilinear corneal contact IOP sensors.

Supplementary Fig. 24 | Fluorescent and optical photographs of cytotoxicity tests on days 2 and 6.

Supplementary Fig. 25 | Survival of cell cultures within 1-7 days.

Supplementary Fig. 26 | Survival and apoptosis rate statistics of cell cultures on days 2 and 6.

Supplementary Fig. 27 | Photographs of rabbit wearing $Ti_3C_2T_x$-SCL.

Supplementary Fig. 28 | Slit-lamp examination photographs of the rabbit eyes after wearing $Ti_3C_2T_x$-SCL.

Supplementary Fig. 29 | White light photos of the rabbit eyes after wearing $Ti_3C_2T_x$-SCL.

Supplementary Fig. 30 | Current dynamic response of temperature sensors during slow temperature rise.

Supplementary Fig. 31 | The sensitivity change rate of $Ti_3C_2T_x$-SCL versus temperature over the IOP range of 0-50 mmHg.

Supplementary Fig. 32 | Photograph of rat skull with motor and sensory cortices.

Supplementary Fig. 33 | Photograph of the sciatic nerve in a rat.

Supplementary Fig. 34 | Potential signals from the somatosensory cortex collected during intraocular injection of saline into the rat eye.

Supplementary Fig. 35 | Stimulation of the somatosensory cortex by impulse signals generated by the neuroprosthetic contact lens in response to abnormal intraocular pressure in the rat and the corresponding potential signals collected in the somatosensory cortex.

Supplementary Fig. 36 | Stimulation of the somatosensory cortex by impulse signals generated by the neuroprosthetic contact lens in response to abnormal intraocular pressure in the rat and the corresponding potential signals collected in the leg.

Supplementary Fig. 37 | Stimulation of the motor cortex by impulse signals generated by the neuroprosthetic contact lens in response to abnormal intraocular pressure in the rat and the corresponding potential signals collected in the leg.

Supplementary Fig. 38 | Circuit boards for data acquisition, processing and generation of corresponding stimulus signals.

Supplementary Fig. 39 | Different levels of the potential signals collected in the somatosensory cortex when the rats were in different IOP ranges.

Supplementary Fig. 40 | Corresponding signals obtained on the ipsilateral gastrocnemius muscle by stimulating the rat sciatic nerve at different frequencies with a current of 0.1 mA.

Supplementary Fig. 41 | Pictures of the corresponding flexor responses of the rat leg when the intraocular pressure is in different horizontal ranges.

Supplementary Tables:

Supplementary Table 1 | Weight comparison of $Ti_3C_2T_x$-SCL with commercial transparent and color contact lenses.

Supplementary Table 2 | Performance comparison with reported articles.

Supplementary Table 3 | Standard value, average deviation and accuracy of the $Ti_3C_2T_x$ -SCL at 0.2 mmHg.

Supplementary Table 4 | Maximum measurement error, minimum measurement error and average measurement error at 0.05, 0.1, 0.15, 0.2 and 0.25 mmHg.

Supplementary Table 5 | Value-added rate statistics table.

Supplementary Table 6 | Survival index statistical table of cell in media containing $Ti_3C_2T_x$-SCL within 7 days.

Supplementary References 1–17

Supplementary Methods.

1 Material characterization

The morphology of 2D $Ti_3C_2T_x$ MXene was observed using field emission scanning electron microscopy (FESEM, JOEL JSM-7500F). The crystal structure of the material was characterised by powder X-ray diffraction (XRD, Bruker D8 Advance). The composition and structure of the samples were analysed by Raman (SNFT-ARLAB1000), Fourier transform infrared spectroscopy (FTIR, Nicolet IS10) and X-ray photoelectron spectroscopy (XPS, PHI VersaProbe III), respectively.

2 Device characterization and measurement

The thickness of the electrodes were measured via atomic force microscopy (AFM, Bruker Dimension XR FastScan). A tensile machine (YL-S71) and an electrochemical work station (CHI 760D) were used together to explore the effect of electrode resistance on sensitivity and the difference in sensitivity between rectilinear and serpentine electrodes at different strains. Meanwhile, the temperature sensors were also under test on the same workstation. The performance of pressure sensing based on the wheatstone bridge structure was tested by the 2636B system sourcemeter. UV-visible spectrophotometer (UV-2600i) was used to evaluate the transparency of $Ti_3C_2T_x$-SCL. The total transmittance and diffuse transmittance of the neuroprosthetic contact lens were tested by ultraviolet-visible absorption spectroscopy (UV-3600) in the visible wavelength range. Electrophysiological signals from rats were collected using a PowerLab data acquisition device and LabChart analysis system.

3 Stimulations

Finite element analysis was used to explore the effect of changes in intraocular pressure on corneal-scleral deformation. The eye model was simplified during simulation with COMSOL Multiphysics to include the cornea, which encompasses approximately the anterior 1/6 of the ocular surface, and the sclera, which encompasses approximately the posterior 5/6 of the ocular surface. Corneal radius is 7.8 mm, scleral radius is 12 mm and both are 1 mm thick. Uniform pressures of 0 mmHg, 10 mmHg, 20 mmHg, 30 mmHg, 40 mmHg and 50mmHg were applied to the eyes to establish a steady state study.

Tensile deformation distribution of equal width rectilinear and curved electrodes was studied by utilising COMSOL Multiphysics. In the model, the $Ti_3C_2T_x$ MXene electrode was tightly wrapped by PDMS. Quasi-static analyses were performed during stretching of the model from 0 to 2%.

4 Cell culture and biocompatibility studies:

(1) Cellular resuscitation: ECM-specific medium (Sciencell) were used for HUVEC cell culture. Initially, HUVEC cells were removed from liquid nitrogen and quickly placed in a 37°C water bath; Shake the cryotube gently to dissolve the cryopreservation solution. After lysis, the cells were transferred to a centrifuge tube containing 5 mL of medium and collected by centrifugation at 1000 rpm for 5 min at room temperature. Cells were then suspended in complete medium containing 10% fetal bovine serum and inoculated into petri dishes; After gentle blowing and tapping to mix, they were incubated at 37°C and 5% CO_2 saturated humidity.

(2) Cell passaging: When the density of the cells reached 80%, the cells were passaged. First, the medium was discarded and the cells were washed once with PBS buffer (1X, Biosharp). After that, 1-2 mL of 0.25% Trypsin-EDTA Solution (Biosharp) was added to digest the cells for 1-2 min. The digestion process was complete when the cells were observed under the microscope to separate from each other and to become rounded. Following this, trypsin was quickly discarded and complete medium was added. Blow and tap again to obtain a single cell suspension. The final passages were passed on at a ratio of 1:3, and the culture was expanded at 37°C and 5% CO_2 saturated humidity.

(3) Cell processing: HUVEC cells in logarithmic growth phase with good growth status were inoculated at 5×10^4 cells/well in cell culture 6-well plates and incubated overnight at 37°C in a 5% CO_2 incubator. The cells were subsequently grouped and numbered as a control group and some material ($Ti_3C_2T_x$-SCL) group (1 day, 2 day...), and the $Ti_3C_2T_x$-SCL were spread over the bottom

of a 6-well plate. After the cells were resuspended to 10^5 cells/ml, each group received 500 μL cell suspension into the middle of the material, and each group was cultured for the indicated time and then assayed.

(4) Cellular CCK8 assay: When the cell culture reached the desired time, 50 μl CCK8 (APExBIO) was added to the cell suspension on each well of material and incubated at 37°C for 1h. Afterwards, the above cell suspension was inoculated into cell culture 96-well plates at 100 μL/well. Finally, the absorbance value of each well was determined using an enzyme-linked immunoassay analyser (OD 450, BK-EL10C). These experiments were repeated at least three times.

5 Slit lamp examination of rabbits

Three rabbits experiments mentioned in the paper were conducted with permission from the Hubei Institutional Animal Care and Use Committee (approval number: 202340191). The four-month-old New Zealand rabbits was used in the experiment. Rinse the $Ti_3C_2T_x$-SCL with PBS solution for 3 min before wearing. At the end of the indicated time (1 day, 2 days... respectively.), the contact lens was removed and 2 μL of 2% fluorescent dye was dropped into the conjunctival sac. The ocular surface was evaluated with a slit lamp (SL-15, kova Optimed) and fundus white light photographs were taken, respectively.

Supplementary Notes:

Supplementary Note 1: Calculation.

1.1 The relationship between resistance changes of the strain Arm and IOP changes

Assuming the initial state (P_1) has a curvature radius of r (r_1) for the corneal contact lens, and a change in intraocular pressure (P_2) results in a change in the curvature radius to $r+\Delta r$ (r_2), as illustrated in Fig. S1. Even at a high intraocular pressure (IOP) of 50 mmHg, the maximum corneal deformation is only 1.13% at approximately 5.12 mm from the center of the cornea (Fig. 2b), thus the tensile angle of the strain arm before and after the IOP change can be considered as α. Then the change in strain gauge radius Δd is:

$$\Delta d=(r+\Delta r)\sin\left[\frac{\alpha r}{2(r+\Delta r)}\right]-r\sin\frac{\alpha}{2} \quad (1)$$

When $\Delta r \ll r$, the equation above can be simplified to:

$$\Delta d\approx\left(\sin\frac{\alpha}{2}-\frac{\alpha}{2}\cos\frac{\alpha}{2}\right)*\Delta r \quad (2)$$

The radius change rate of the strain gauge here is

$$\beta=\frac{\Delta d}{d}=\gamma*\Delta r \quad (3)$$

where d is the initial radius of the strain arm and $\gamma=\frac{\left(\sin\frac{\alpha}{2}-\frac{\alpha}{2}\cos\frac{\alpha}{2}\right)}{d}$ is a constant.

Since changes in the IOP will cause curvature changes in the radius of the corneal contact lens, the expression is shown below:

$$\Delta r=\delta*\Delta P \quad (4)$$

where $\Delta P= P_1-P_2$ is the amount of IOP change and δ is a constant that depends on the mechanical properties of the eyeball and corneal contact lens. Therefore, the change in resistance ΔR of the strain arm can be obtained from Eqs. (S3) and (S4):

$$\Delta R=\varepsilon*\Delta P \quad (5)$$

$\varepsilon=\gamma*\delta*GF*R_0$ is a constant and GF is the strain sensitivity factor, so the change in resistance of the strain arm correlates approximately linearly with the change in IOP.

1.2 The relationship between the output voltage in a corneal contact lens based on a Wheatstone bridge circuit and IOP changes

The Wheatstone bridge pressure sensor consists of two strain resistors (R_1 and R_3) and two reference resistors (R_2 and R_4) as shown in Fig. 2f. When excited by a constant current source I, the bridge output U is

$$U=\left(\frac{R_2}{R_1+R_2}-\frac{R_4}{R_3+R_4}\right)*I*R_0 \quad (6)$$

When the intraocular pressure changes, it is assumed that the resistance changes as

$$R_1=R_4=R_0\text{-}\Delta R \quad (7)$$

$$R_2=R_3=R_0+\Delta R \quad (8)$$

where R_0 is the initial resistance, then

$$U=\Delta R*I \quad (9)$$

The equation can be derived from Eqs. (S5) and (S9):

$$U=\varepsilon*\Delta P*I \quad (10)$$

Therefore, the output voltage of the corneal contact lens is proportional to the change in IOP.

1.3 The effect of temperature on the output voltage of $Ti_3C_2T_x$ MXene based Wheatstone bridge pressure sensors.

The inhomogeneity of corneal deformation during IOP changes creates varying energy barriers for electron jumps within each resistor, resulting in different temperature coefficients (k) between resistors at that instant.

$$k\sim R \quad (11)$$

If the temperature is T, then the resistance under pressure of the stretched and reference arms at an intraocular pressure of P are as follows:

$$R_1'=R_4'=R_m \quad (12)$$

$$R_2'=R_3'=R_n \quad (13)$$

The resistance under the combined effect of pressure and temperature is obtained by combining Eqs. (S11), Eqs. (S12) and (S13):

$$R_1=R_4=(1+K_mT)R_m \quad (14)$$

$$R_2=R_3=(1+K_nT)R_n \quad (15)$$

Thus, the output voltage U_ξ of the Wheatstone bridge corneal contact lens at temperature T and intraocular pressure P is

$$U_\xi=\left[\frac{(1+K_nT)R_n}{(1+K_mT)R_m+(1+K_nT)R_n}-\frac{(1+K_mT)R_m}{(1+K_nT)R_n+(1+K_mT)R_m}\right]*U_0$$

$$=\left[\frac{(1+K_\mathrm{n}T)R_\mathrm{n}-(1+K_\mathrm{m}T)R_\mathrm{m}}{2[(1+K_\mathrm{m}T)R_\mathrm{m}+(1+K_\mathrm{n}T)R_\mathrm{n}]}\right]*U_0$$

$$=\frac{K_\mathrm{n}R_\mathrm{n}-K_\mathrm{m}R_\mathrm{m}}{2(R_\mathrm{m}+R_\mathrm{n})}*T*U_0 \qquad (16)$$

where U_0 is the input voltage and Eq. (S16) is obtained by neglecting the higher order terms. It can be seen that the temperature affects the output voltage during pressure variations.

Supplementary Note 2: Characterization of $Ti_3C_2T_x$ MXene.

The crystal structure of the $Ti_3C_2T_x$ MXene was analysed by x-ray diffraction as shown in Fig. S7. The multi-layered $Ti_3C_2T_x$ MXene etched by the mixed acid has an accordion-like structure, and thus diffraction peaks with different angles appear in the spectrum. In contrast, the mono-layered $Ti_3C_2T_x$ MXene has almost no high angle diffraction peaks due to the embedding of LiCl in the layers of the multi-layered $Ti_3C_2T_x$ MXene, which separates it into individual nanosheets[1]. The smaller angle of the (002) diffraction peak for the mono-layered $Ti_3C_2T_x$ MXene (7.25°) than for the multi-layered $Ti_3C_2T_x$ MXene (8.34°) also suggests a larger layer spacing. These phenomena indicate that the $Ti_3C_2T_x$ MXene changes from multilayer structure to single layer. In order to analyse the composition and structure, the $Ti_3C_2T_x$ MXene was tested by Raman spectroscopy (Fig. S8)[2]. The Raman spectra of 785 nm laser excitation can clearly reflect the vibrations of the three regions of $Ti_3C_2T_x$ MXene. The peak located at 202 cm^{-1} is flake region consisting of E_g (Ti, C, O) and A_{1g} (Ti, C, O) modes. The peaks in the region 230 cm^{-1} ~ 470 cm^{-1} are attributed to the vibrations of functional groups on the surface of Ti atoms. The region between 580 cm^{-1} ~ 730 cm^{-1} represents the carbon vibration. Fourier transform infrared spectroscopy (FTIR) was carried out on $Ti_3C_2T_x$ MXene as can be seen from Fig. S9. The FTIR analysis revealed characteristic absorption peaks of C=O at 1167 cm^{-1} and stretching vibrational absorption peaks of the -OH group from 3567 cm^{-1} to 3776 cm^{-1} [3]. The hydrophilic functional groups on the surface of $Ti_3C_2T_x$ MXene facilitate device preparation. The composition of chemical bonds in the $Ti_3C_2T_x$ MXene was analysed by x-ray photoelectron spectroscopy based on the result of Fig. S10. As expected, peaks of F 1*s*, O 1*s*, Ti 2*p* and C 1*s* appear at 658, 530, 456 and 282 eV, respectively, further indicating the elemental composition of the $Ti_3C_2T_x$ MXene[4]. Due to spin orbital splitting in the $Ti_3C_2T_x$ MXene, Ti 2*p* produces three double peaks, the Ti-C peaks at 458.1 eV and 454.0 eV, the Ti-F peaks at 455.4 eV and 461.6 eV, and the T-O peak at 406.0 eV[5]. C 1*s* produced an O-C=O characteristic peak at 288.0 eV, a C-O characteristic peak at 286.0 eV, a C-C characteristic peak at 283.8 eV, and a Ti-C characteristic peak at 281.0 eV. O 1*s* bonded to Ti (T-O) at 528.8 eV and 529.7 eV, to O at 531.3 eV (C-O) and 532.4 eV (C=O), and to H (-OH) at 533.2 eV, respectively[6]. The abundance of oxygen-containing functional groups on the surface of $Ti_3C_2T_x$ MXene improves electrical conductivity while imparting excellent hydrophilicity. The contact angle between the $Ti_3C_2T_x$ MXene film and deionized water was only 20.9° (Fig. S11), indicating its

suitability for flexible processing.

Supplementary Note 3: Comparisons of the $Ti_3C_2T_x$-SCL devices with commercial corneal contact lenses.

Haze is an important parameter for corneal contact lenses, and an increase in haze will result in a decrease in gloss as well as transparency and especially imaging. Haze is the cloudy or cloudy appearance of the interior or surface of a transparent or semi-transparent material due to light diffusion, expressed as a percentage of the ratio of the diffused luminous flux to the luminous flux through the material. Therefore, the transmittance and haze of the neuroprosthetic contact lens were tested as 94.7% and 3.17% at 550 nm by ultraviolet-visible absorption spectroscopy (UV-3600), respectively.

10 random devices were weighed and a mean value of 0.0314 g was obtained, which shows lighter weight than 0.041 g for transparent contact lenses and 0.364 g for color contact lenses from Bausch & Lomb, 0.0355 g for transparent contact lenses and 0.0392 g for color contact lenses from Cooper Optics, and 0.0339 g for transparent contact lenses and 0.0370 g for color contact lenses from Hydron (Table S1).

Supplementary Note 4: Analog intraocular pressure testing platform.

A test platform was constructed to evaluate the performance of sensors that measure intraocular pressure (IOP), including a simulated eyeball, a pressure regulation system, and a sensor signal test system (Fig. S15). The eyeball wall consists of a stainless steel chamber designed to mimic the sclera and a bionic cornea that enables the monitoring of pressure and temperature, while the contents are anhydrous ethanol and connected to an infusion bag that hangs from a motorized sliding table. The motorized sliding table can be programmed to control pressure changes within the bionic eye through the pressure adjustment system, whilst a manometer attached to the other end of the stainless steel chamber measures the pressure on the cornea in real time. In addition, the source meter in the pressure feedback system is connected to the sensor on the bionic cornea, where one channel provides a constant current to the sensor and the other channel is used to detect the output voltage value in response to the intraocular pressure.

Supplementary Note 5: Performance indicators of the $Ti_3C_2T_x$-SCL.

5.1 The smallest detection limit is the lowest limit at which the sensor can accurately reflect what is being measured. The $Ti_3C_2T_x$ -SCL was tested 80 times at different small pressures, and the output voltage changes at 0.025 mmHg and 0.05 mmHg appearing partially overlap, with a large gap between the output voltage changes at 0.05 mmHg and 0.1 mmHg as shown in Fig. S18a, b. Further statistical analysis indicates that the maximum relative error of the output voltage change at 0.025 mmHg is larger at 55.03%, while it is only 16.95% and 6.15% for 0.05 mmHg and 0.1 mmHg (Fig. S18c). The large relative error prevents the sensor from resolving pressures of 0.025 mmHg, so the smallest detection limit of $Ti_3C_2T_x$ -SCL is 0.05 mmHg. To verify the consistency, 10 devices were tested at a pressure of 0.05 mmHg for 80 cycles each, and Fig. S18d exhibits that the average measurement error is less than 3.6%, which demonstrates the $Ti_3C_2T_x$ -SCL offers the smallest detection limit of 0.05 mmHg.

5.2 The measurement error of the sensor is mainly divided into systematic error and random error, where the systematic error is caused by the inherent characteristics of the sensor itself or imperfections in the manufacturing process, and the random error is caused by external environmental factors and changes in the measurement conditions, which are calculated as follows:

$$E = |V_m - V_s| \quad (17)$$

Where E is the error of the sensor and V_m and V_s are the measured and standard values at the same pressure.

To obtain the measurement errors of the IOP stress sensor, 8 devices were randomly selected and tested at pressures of 0.05, 0.1, 0.15, 0.2 and 0.25 mmHg, and 80 points were statistically analyzed at each pressure, as reported in Table S4. Fig. S21a, b shows that the measurement errors of all devices are in good agreement at the same pressure. Fig. S21c, d further indicates that the average measurement error of the 8 devices is 0.030 mV at a pressure of 0.05 mmHg, 0.030 mV of 0.1 mmHg, 0.028 mV of 0.15 mmHg, 0.026 mV of 0.20 mmHg, 0.031 mV of 0.25 mmHg, and that the average measurement error of IOP strain sensor is 0.029 mV.

5.3 Accuracy is the difference between the measurement result and the standard value, and is calculated by the following formula:

$$A=\frac{V_m}{V_s} \qquad (18)$$

where A represents the accuracy of the device, V_m is the average deviation and V_s is the standard value.

We performed 80 tests on each of the 15 devices at a pressure of 0.2 mmHg and calculated the corresponding standard values, average deviations and accuracies, as listed in Table S3 and Fig. S20, showing the accuracy of the $Ti_3C_2T_x$ -SCL at 0.2 mmHg pressure of 1.075% as a result of statistical analysis.

Supplementary Note 6: Performance testing of rectilinear corneal contact IOP sensors.

$Ti_3C_2T_x$-SCL with a linear shape of the passive gauges were re-prepared and performed a full set of performance tests to further verify the effect of electrode shape on the sensitivity. The pressure was applied to the cornea at a speed of 1x with a pause of 10 s for every change of 6.25 mmHg, and then reduced to the initial state in the same way when reached 50 mmHg, and the measured output voltage change curve was in the form of a step, which was able to distinguish the change of the pressure accurately (Fig. S23a). Fig. 23b illustrates a good linear relationship between the output voltage variation and the pressure value, with a sensitivity of 3.8 mV $mmHg^{-1}$ (R^2= 0.99345) in the pressure rise phase and 4.39 mV $mmHg^{-1}$ (R^2= 0.99727) in the pressure fall phase, which is lower than that of the $Ti_3C_2T_x$-SCL with a serpentine resistance. The output voltage change curves at 1x, 3x and 5x speeds were synchronized with the pressure change curves without significant delay, indicating that the sensor can monitor rapid IOP changes, such as sudden drops in ambient temperature, extreme sports, external impacts, and so on (Fig. S23c). At the same time, the output voltage change can quickly respond to the pressure change in different voltage amplitude and long time cycling process, respectively (Fig. S23d-e).

Supplementary Note 7: Comparison with reported articles.

A variety of materials and methods have been used to fabricate smart contact lenses for monitoring IOP, but their clinical application poses several challenges in areas such as measurement range and sensitivity as detailed in Fig. 2i-j and Table S1. Corneal contact lenses prepared by embedding platinum-titanium (Pt-Ti) strain gauges into polymer substrates achieved sensitivities of 0.008 mV $mmHg^{-1}$, 0.02 mV $mmHg^{-1}$ and 0.109 mV $mmHg^{-1}$ in the ranges of 17-29 mmHg, 10-30 mmHg and 11-30 mmHg, respectively[7-9]. In further work, Dou et al. constructed a Wheatstone bridge circuit with platinum-titanium strain gauges, which resulted in an increase in sensitivity of 0.29 mV $mmHg^{-1}$ (15-35 mmHg)[10]. Compared to bulk phase materials, Xu and Liu et al. utilized graphene nanosheets with superior piezoresistive properties to fabricate corneal contact lens-type IOP sensors which combined with the structural design of Wheatstone bridge circuits to achieve high sensitivities of 0.15 mV $mmHg^{-1}$ and 1.047 mV $mmHg^{-1}$ in the ranges of 10-35 mmHg and 10-50 mmHg[11, 12]. Meanwhile, Zhang et al. presented a contact lens ophthalmotonometer based on graphene woven fabrics with an average resolution of 6.8% $mmHg^{-1}$ over a variation range of 0-10 mmHg[17]. Fan et al. also reported an IOP sensor based on a Wheatstone bridge structure prepared using a mixture of reduced graphene oxide and carbon nanotubes (GO-CNTs) with the detection range of 9-34 mmHg and the detection sensitivity of 0.036 mV $mmHg^{-1}$ [13]. Capacitive corneal contact lens sensors with a higher detection range than resistive sensors have been successively reported. Chen et al. developed capacitive corneal contact lenses using Cu electrodes to extend the detection range to 5-40 mmHg (0.023 MHz $mmHg^{-1}$)[15], and Kim et al. even achieved a wide detection range of 0-50 mmHg (2.64 MHz $mmHg^{-1}$) with a hybrid electrode of graphene and AgNWs[14]. Surprisingly, Laukhin et al. broke through the ultra-wide detection range of 0-52 mmHg (1.5 Ω $mmHg^{-1}$) by utilizing a flexible conductive all-organic bilayer film (COBL) with ultra-high piezoresistive properties[16].

Supplementary Note 8: Exploration of intraocular pressure sensorimotor circuits in the rat.

We hierarchically explored the neurotransmission process from the eye to the leg in rats by surgically exposing the S_1 somatosensory cortex and M_1 motor cortex in the nociceptors (Fig. S32) and the sciatic nerve in the leg (Fig. S33), respectively. First, saline was injected into the eyeballs of rats to raise the intraocular pressure, at which point no significant signal change from the somatosensory cortex was observed as observed from Fig. S34, suggesting weak neural oscillations between the eye and the brain. A simulated eyeball was used to create an IOP abnormality and stimulate the rat's somatosensory cortex by generating impulse signals through the neuroprosthetic contact lens, recording consistent potential changes with the stimulation signal in this cortex (Fig. S35), suggesting that our device can modulate neural oscillations between the eyeball and brain, allowing rats to feel the IOP change. Meanwhile, potential changes corresponding to the stimulus signals were similarly captured in the motor cortex and gastrocnemius muscle when neuroprosthetic contact lens detected IOP abnormalities as shown in the main text Fig. 5a III and Fig. S36, 37, respectively, confirming the feedback of sensory signals by the rat organism. To produce graded sensory and motor feedback, IOP levels were graded, i.e., 0-9 mmHg for low IOP levels, 10-21 mmHg for normal IOP levels, greater than 21 mmHg for high IOP levels and categorized into three grades of 22-30 mmHg, 31-40 mmHg, and 41-50 mmHg. When IOP abnormalities were created using a simulated eyeball and stimulated in the rat somatosensory cortex by generating pulsed signals through a neuroprosthetic contact lens, graded potential changes were detected in the somatosensory cortex (Fig. S39), suggesting that our device modulates neural oscillations between the eyeball and the brain to make IOP changes perceptible to the rat. Finally, the sciatic nerve was stimulated at different frequencies with a current of 1 mA to simulate different rates of intraocular pressure change, where acquired signals at the gastrocnemius corresponded to the stimulated signals, and the rat's leg twitching accelerated as the frequency of stimulation increased (Fig. S40).

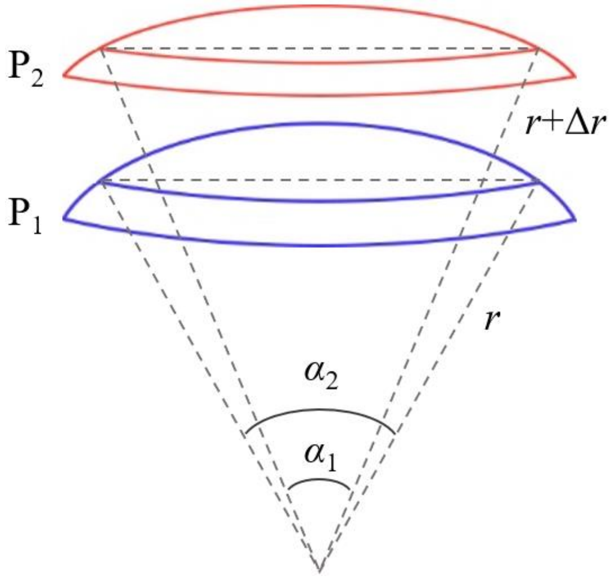

Supplementary Fig. 1. The curvature changes of corneal contact lenses worn by the eye during an increase in intraocular pressure from P_1 to P_2.

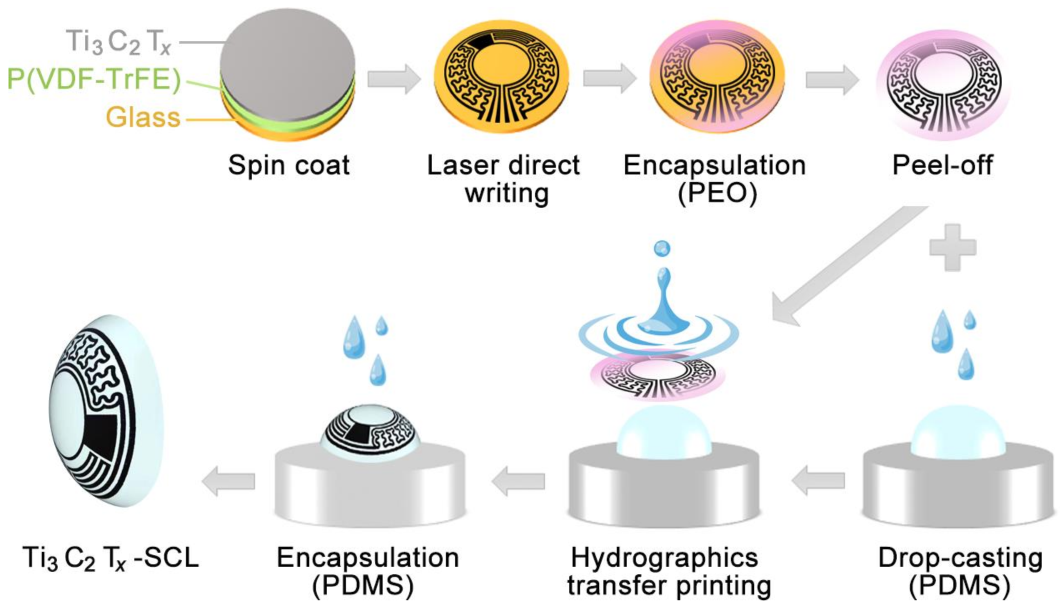

Supplementary Fig. 2. Schematic diagram of the $Ti_3C_2T_x$-SCL preparation process.

The patterned electrodes are realized by laser direct writing technology and then peeled off together with the PEO film, which was possible due to the high ductility of the $Ti_3C_2T_x$ MXene nanosheets and the hydrophobicity of the P(VDF-TrFE) film. The electrodes were then transferred to the PDMS substrate during the dissolution of PEO by deionized water.

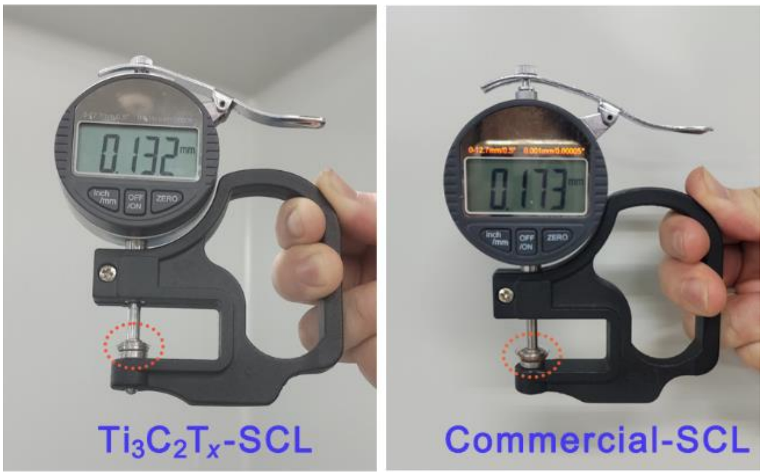

Supplementary Fig. 3. Thickness measurement of $Ti_3C_2T_x$-SCL (left) and commercial-SCL (right).

The center thickness of $Ti_3C_2T_x$-SCL was measured with a film thickness gauge and was between 130 ~ 140 μm, which meets the requirements for commercial lenses (<173 μm) and ensures the uniformity of the device.

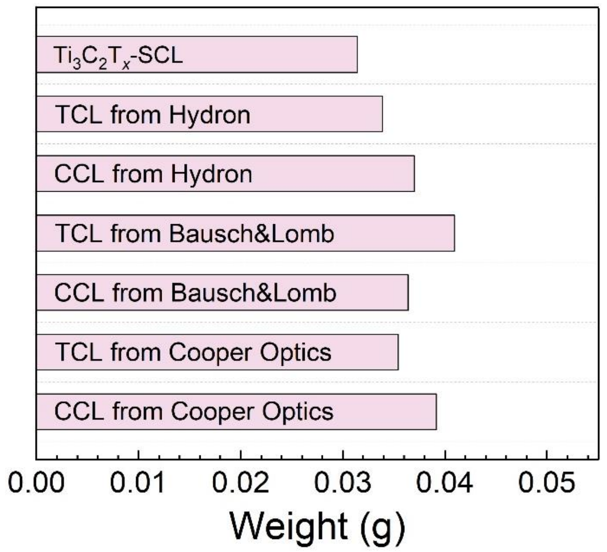

Supplementary Fig. 4. Average weight of $Ti_3C_2T_x$-SCL and weight comparison with commercial transparent contact lenses (TCL) and color contact lenses (CCL).

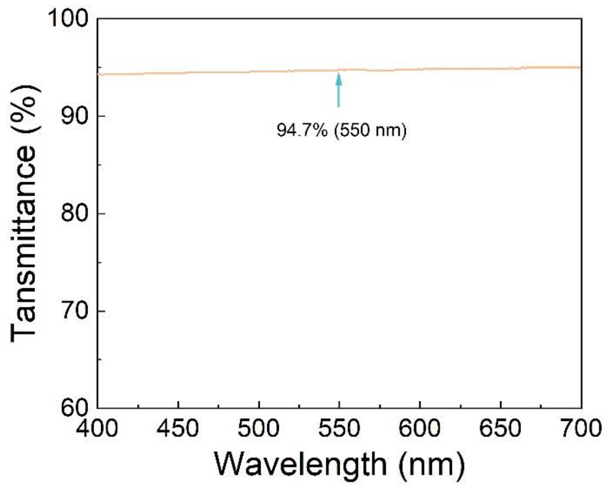

Supplementary Fig. 5. Transmittance of $Ti_3C_2T_x$-SCL.

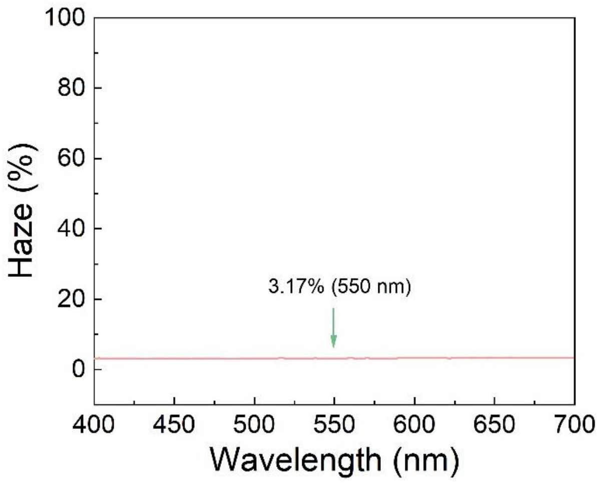

Supplementary Fig. 6. Haze of $Ti_3C_2T_x$-SCL.

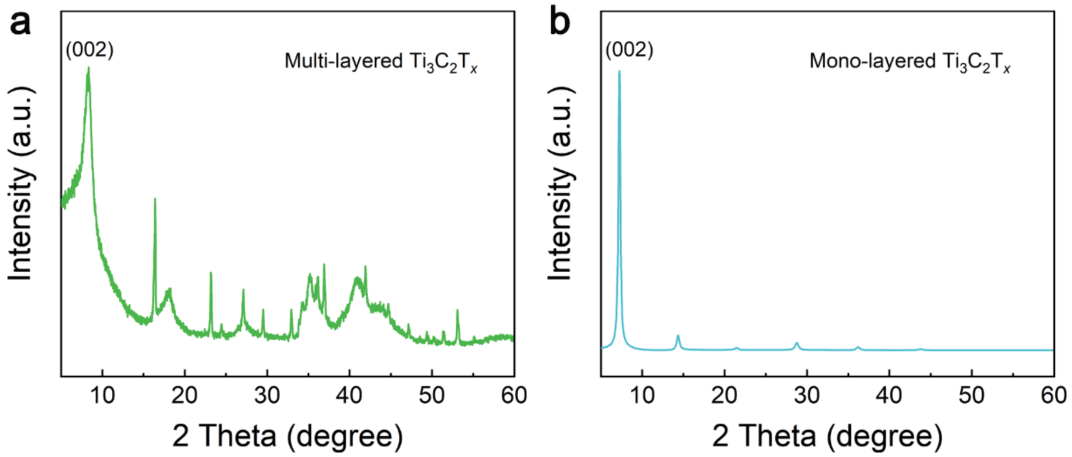

Supplementary Fig. 7. XRD pattern. a multi-layered $Ti_3C_2T_x$ MXene. **b** mono-layered $Ti_3C_2T_x$ MXene.

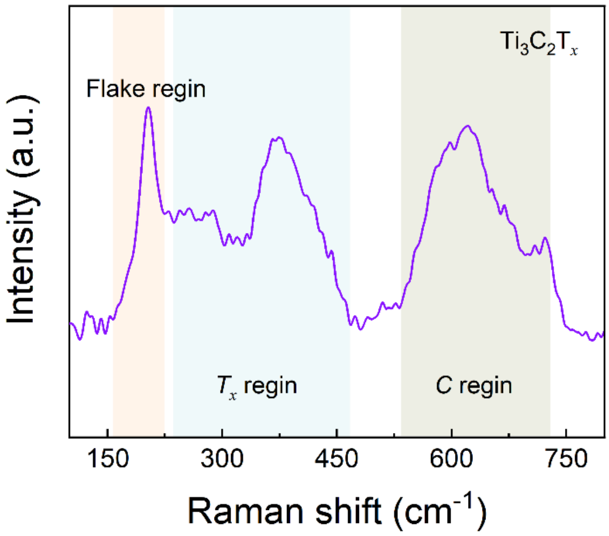

Supplementary Fig. 8. Raman spectra of $Ti_3C_2T_x$ MXene.

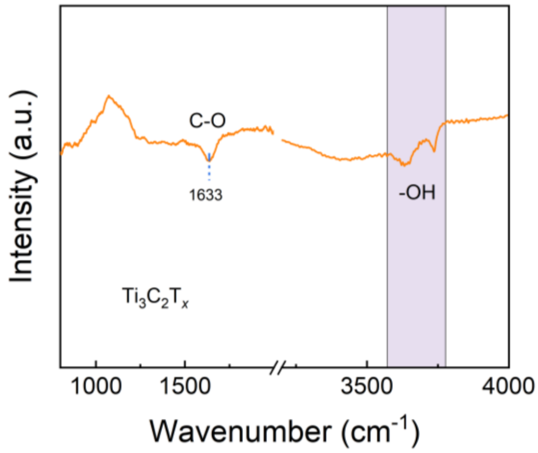

Supplementary Fig. 9. FTIR of $Ti_3C_2T_x$ MXene.

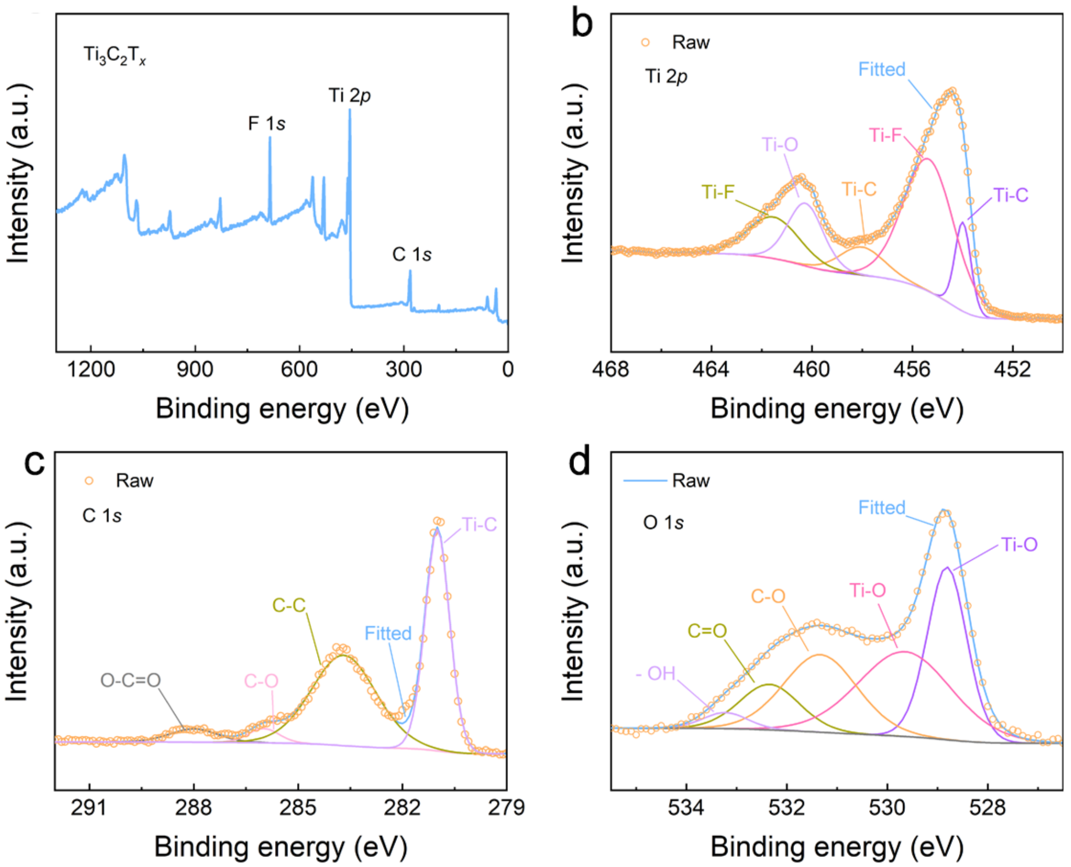

Supplementary Fig. 10. XPS spectra and high magnification. **a** $Ti_3C_2T_x$ MXene. High magnification of **b** Ti 2*p*, **c** C 1*s* and **d** O 1*s* elements.

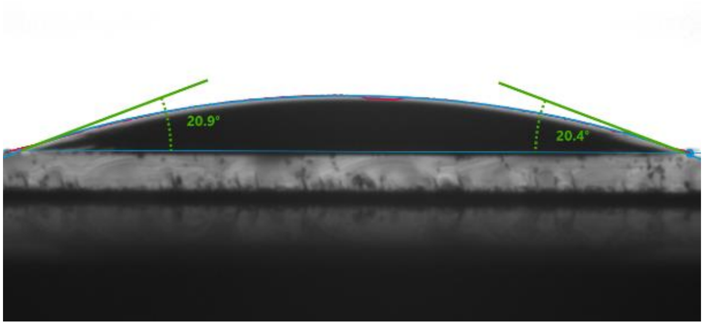

Supplementary Fig. 11. Contact angle test of $Ti_3C_2T_x$ MXene film with water.

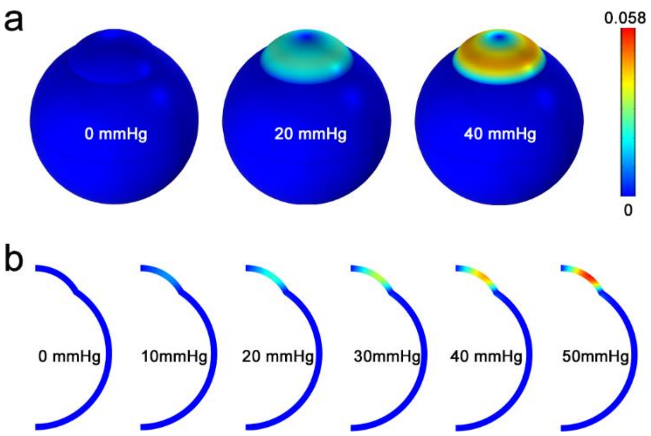

Supplementary Fig. 12. Simulation of eyeball deformation under different IOP. a The Surface stress distribution at 0, 20, and 40 mmHg pressures. **b** Cross-sections of the cornea at six different IOP levels: 0, 10, 20, 30, 40 and 50 mmHg, revealing the location of maximum corneal deformation and the change in pressure distribution across the cornea during IOP elevation.

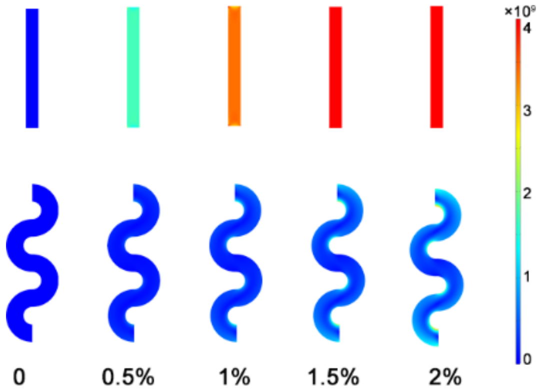

Supplementary Fig. 13. Finite element analysis of rectilinear and serpentine electrodes under 0%, 0.5%, 1%, 1.5% and 2% strain.

The simulation shows that the rectilinear electrode is subjected to much greater stress than the serpentine electrode at the same strain, which leads to the slippage of the $Ti_3C_2T_x$ MXene nanosheets and consequently increases the electrode resistance. Thus the active gauges are designed as a rectilinear and the passive gauges as a serpentine.

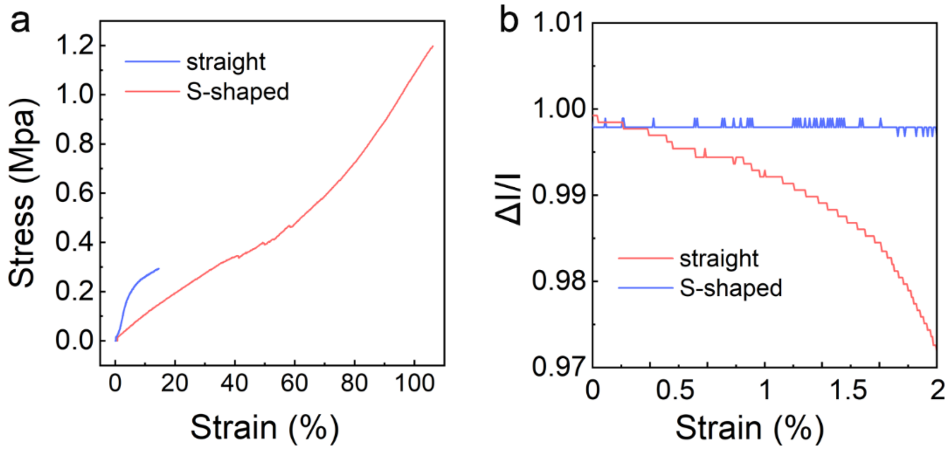

Supplementary Fig. 14. Comparison of serpentine and rectilinear electrodes. a Stress-strain curves. **b** Current variation versus tensile strain from 0 to 2% (The test voltage is 1 V).

Compared to the serpentine electrode, the current of the rectilinear electrode drops sharply with increasing strain (Fig. 2h and Fig. S10b) which is due to the fact that the rectilinear electrode experiences more stress for the same strain, resulting in greater slip distance of the $Ti_3C_2T_x$ MXene nanosheets in the electrode and hence greater change in resistance.

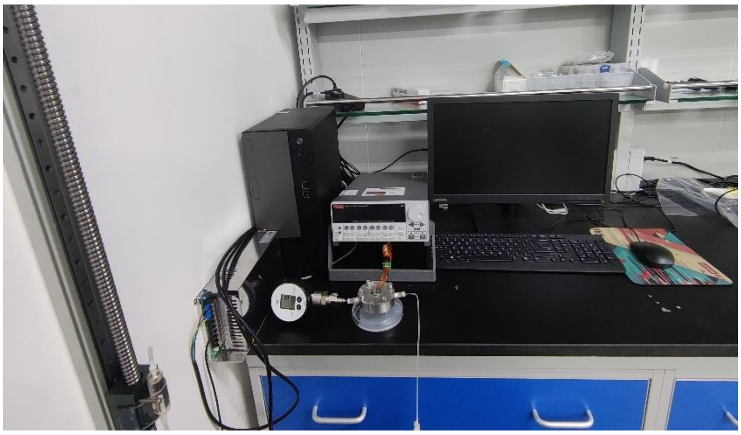

Supplementary Fig. 15. Digital photograph of a simulated IOP testing platform containing bionic eyeball, motorized sliding table with program control system, manometer and source meter.

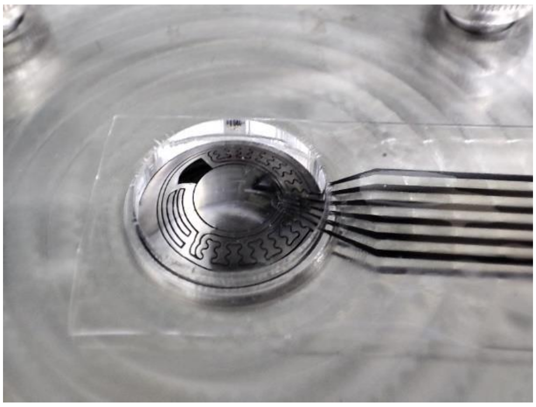

Supplementary Fig. 16. Digital photograph of the bionic eyeball consisting of a stainless steel chamber and a intraocular pressure sensor based on full $Ti_3C_2T_x$ MXene.

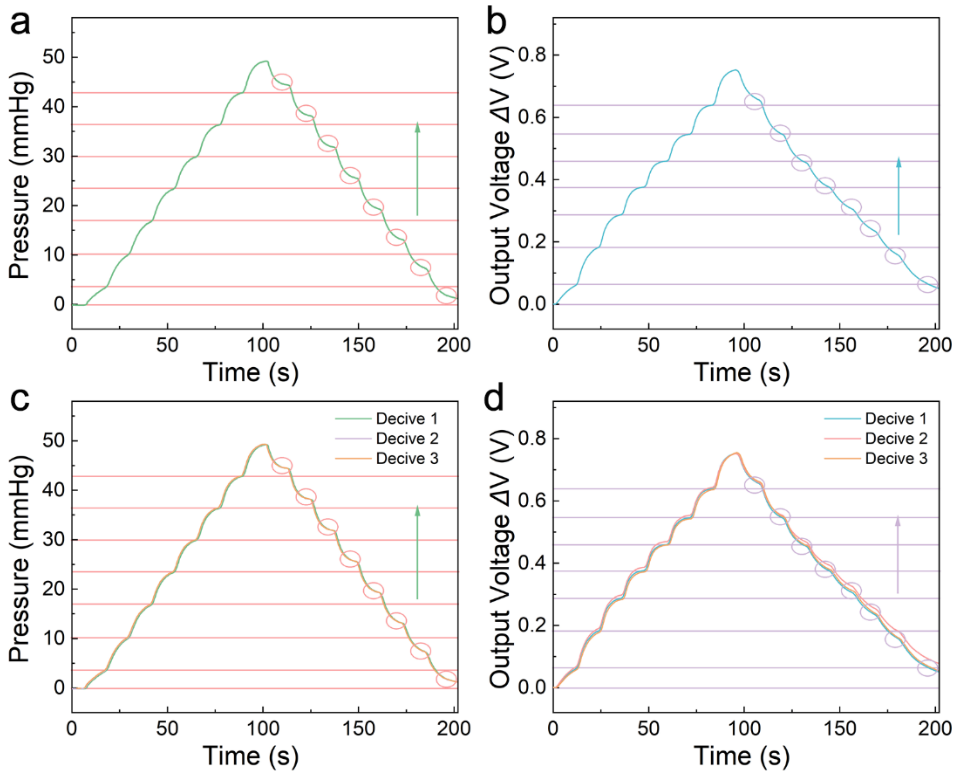

Supplementary Fig. 17. Static response curve for $Ti_3C_2T_x$-SCL. a, b Pressure change and static response of single $Ti_3C_2T_x$-SCL and **c, d** three separate devices increasing from 0 mmHg to 50 mmHg and then decreasing to 0 mmHg.

To apply stepped pressure to the bionic eye, a program was written to stop the motorized sliding table for 10 s (the maximum dwell time allowed by the program) for each 6.25 mmHg change in pressure. The residence time of the motorized sliding table is too short to be sufficient for the anhydrous ethanol to reach equilibrium, leading to a certain degree of difference in pressure during rise and fall. We retested the static response of the single $Ti_3C_2T_x$ -SCL several times, and found our IOP sensors could capture the trends during rise and fall and reach balance, as shown in Fig. S17a and Fig. S17b (modified Figure 3b), which clearly displayed that the pressure and output voltage trends are the same during the rise and fall and finally become stable, allowing the $Ti_3C_2T_x$ IOP strain sensor to accurately obtain an IOP value. Moreover, to verify the repeatability and uniformity of the IOP sensors, the static response of three separate $Ti_3C_2T_x$ -SCL was also carried out (Fig. S17c and Fig. S17d), which shows negligible changes in pressure and output voltage, suggesting that the $Ti_3C_2T_x$ -SCL could be used for IOP monitoring.

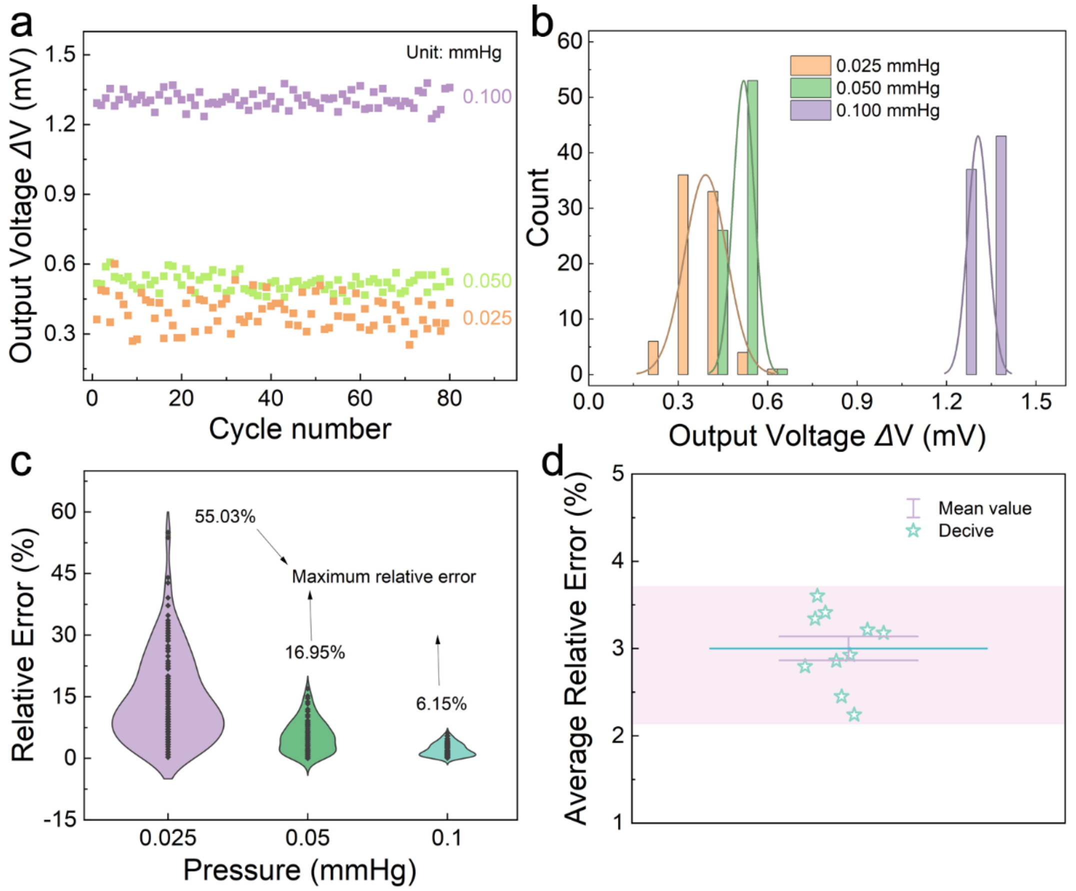

Supplementary Fig. 18. The smallest detection limit of the $Ti_3C_2T_x$-SCL. a The output voltage variations of the $Ti_3C_2T_x$ -SCL at 0.025, 0.05 and 0.1 mmHg, respectively, and **b** the corresponding statistical distributions. **c** Relative errors in the output voltage changes of the $Ti_3C_2T_x$ -SCL at 0.025, 0.05 and 0.1 mmHg. **d** The average measurement error of the output voltage change obtained from 10 devices at a pressure of 0.05 mmHg.

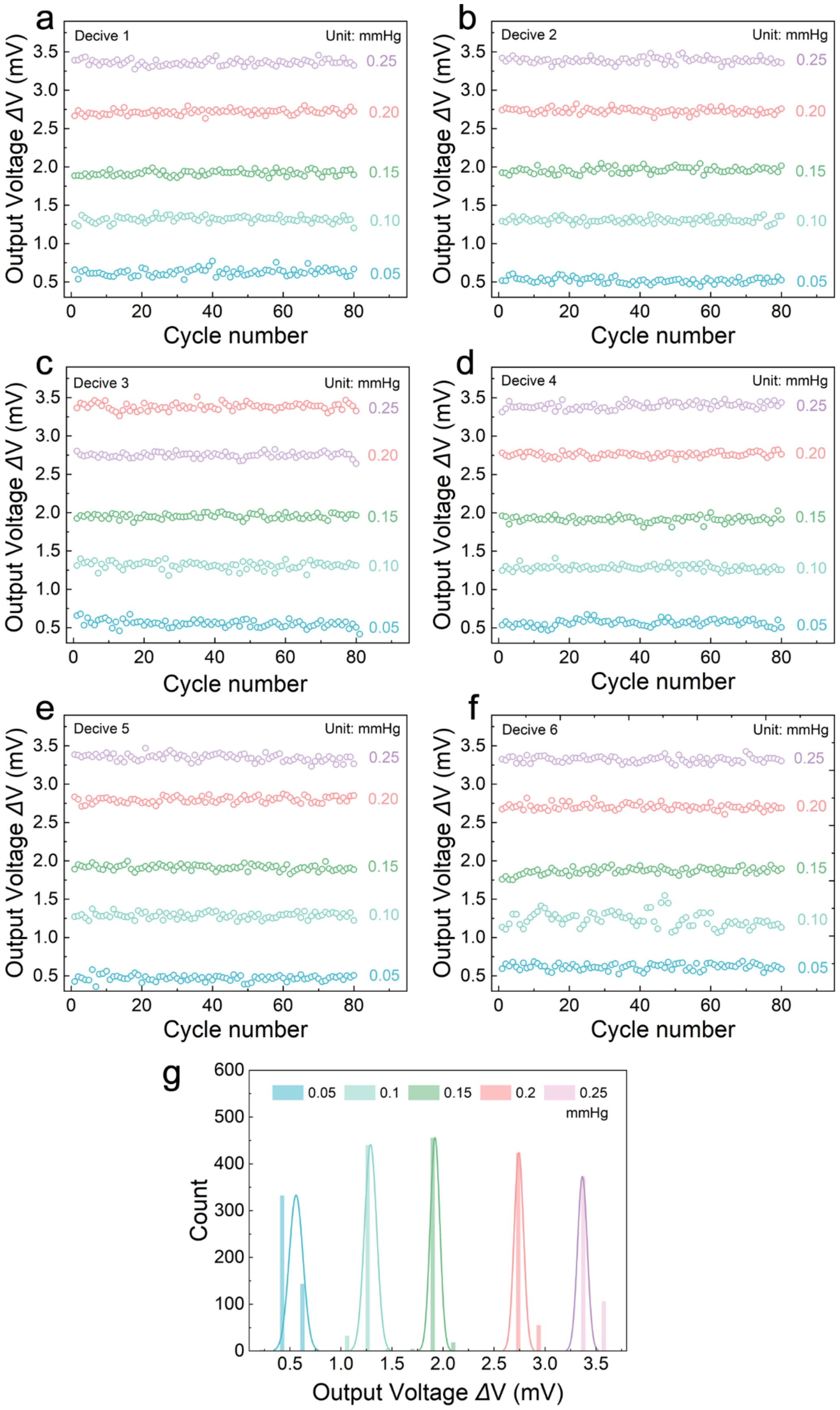

Supplementary Fig. 19. a-f Output voltage variations and **g** statistical distribution of the $Ti_3C_2T_x$ -SCL at 0.05, 0.1, 0.15, 0.2 and 0.25 mmHg, respectively.

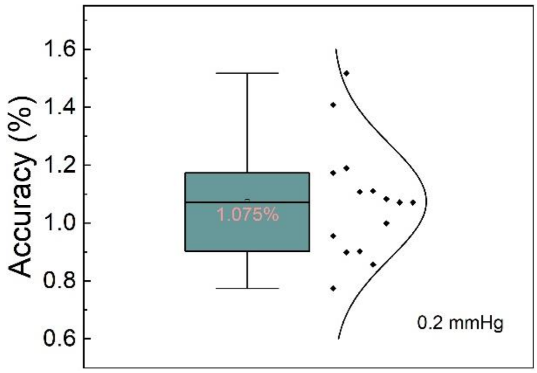

Supplementary Fig. 20. Statistical analysis of the $Ti_3C_2T_x$ -SCL accuracy at 0.2 mmHg.

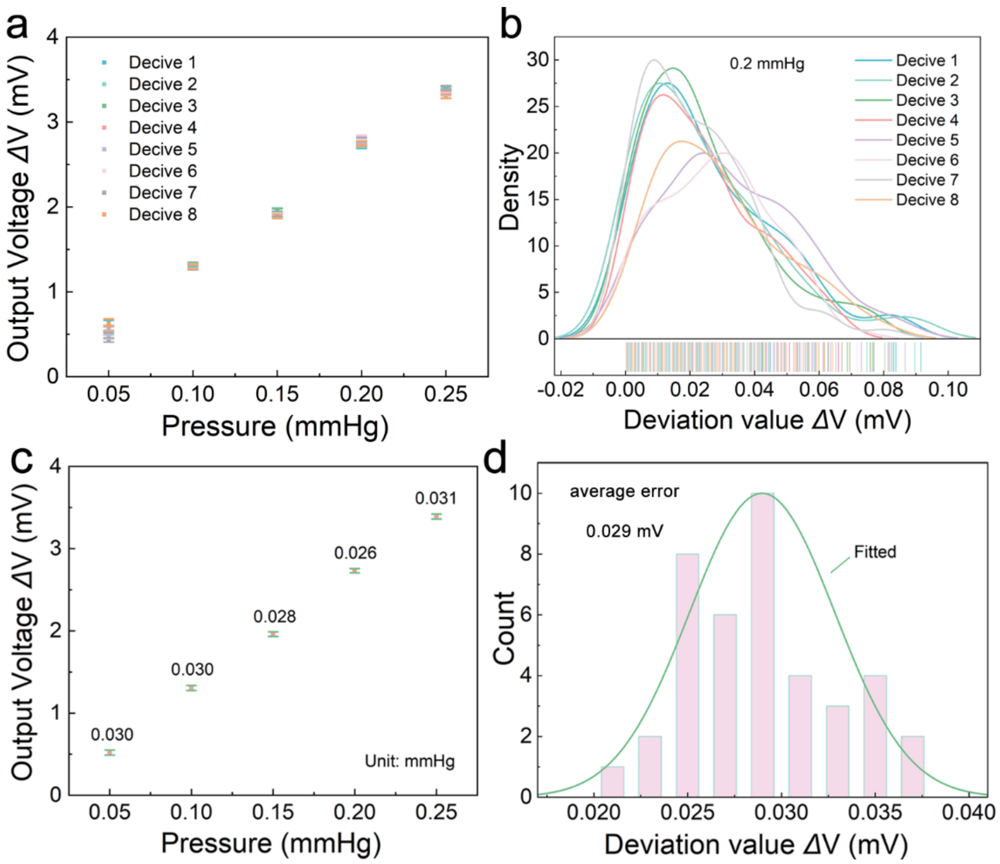

Supplementary Fig. 21. The measurement error of the $Ti_3C_2T_x$-SCL. a Average output voltage variations and average measurement errors for each IOP stress sensors at pressures of 0.05, 0.1, 0.15, 0.2, and 0.25 mmHg, respectively as well as **b** statistical distributions of the measurement errors for each device at a pressure of 0.2 mmHg. **c** Average output voltage variations and average measurement errors of the IOP stress sensors as well as **d** the statistical distribution of the average measurement error at all pressures at pressures of 0.05, 0.1, 0.15, 0.2, and 0.25 mmHg, respectively.

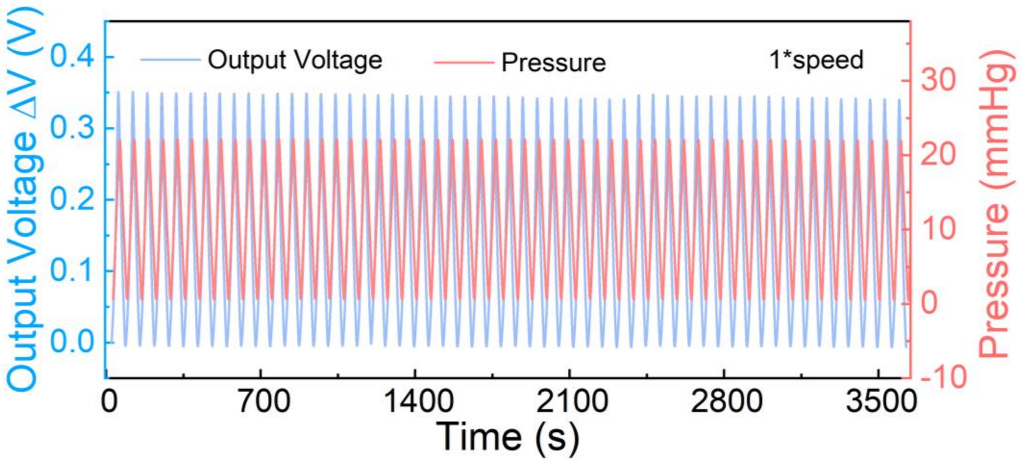

Supplementary Fig. 22. Cycling test at 1x speed in the range of 0-21 mmHg.

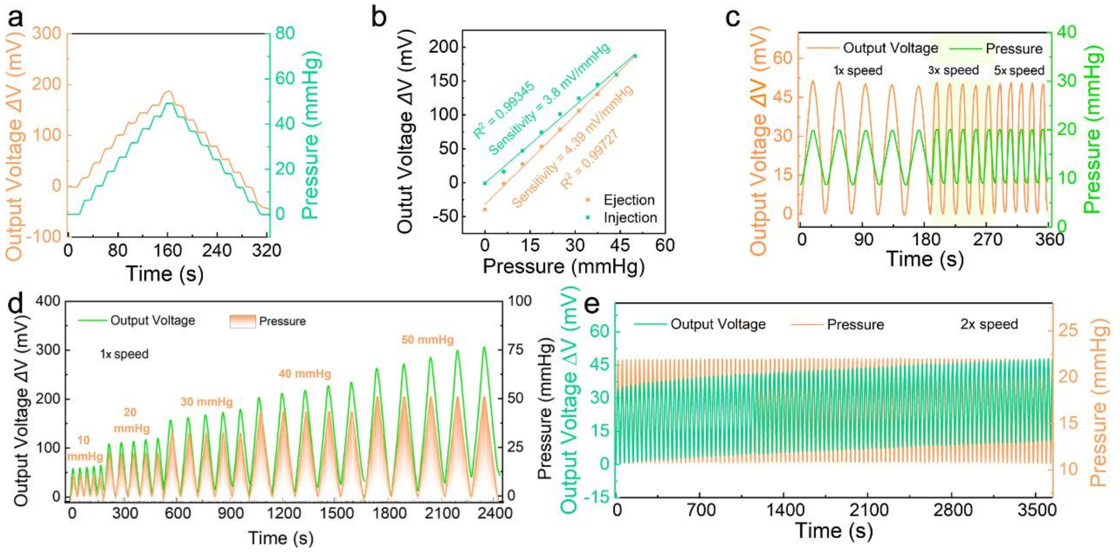

Supplementary Fig. 23. Performance testing of rectilinear corneal contact IOP sensors. a Static response test with 2 mmHg pressure change steps at 1x speed. **b** Fitting curve between output voltage change and pressure. **c** Cyclic testing at different speeds (1x, 3x and 5x), **d** under different pressure amplitudes (0-10, 0-20, 0-30, 0-40 and 0-50 mmHg) and **e** in the pressure range of 10 to 21 mm Hg for 1 h, respectively.

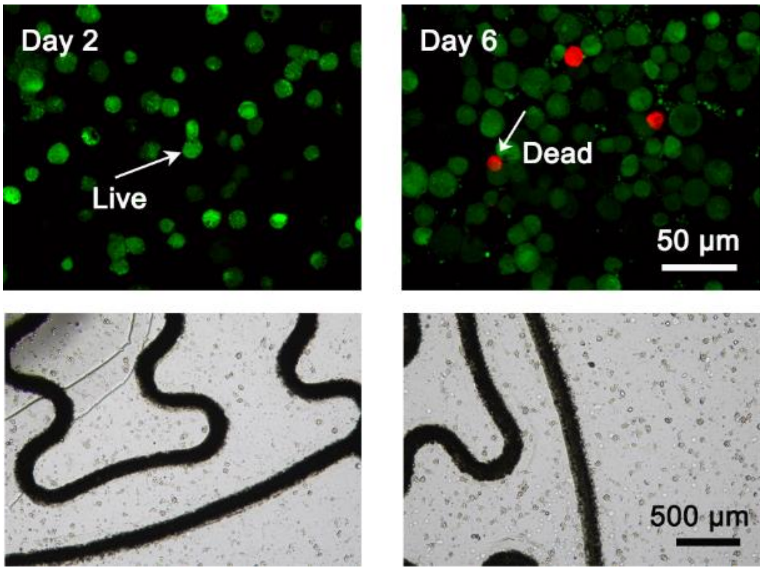

Supplementary Fig. 24. Fluorescent and optical photographs of cytotoxicity tests on days 2 and 6.

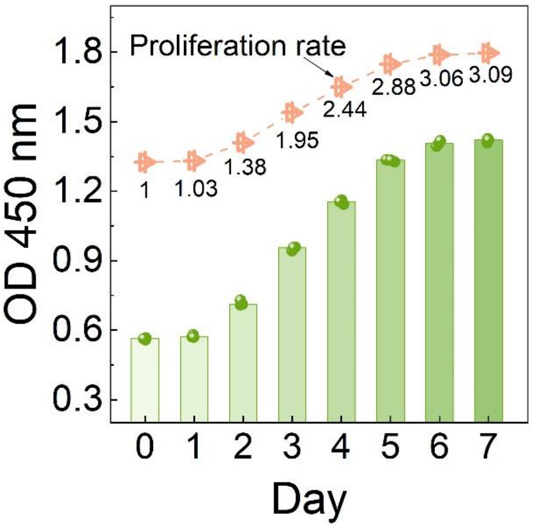

Supplementary Fig. 25. Survival of cell cultures within 1-7 days.

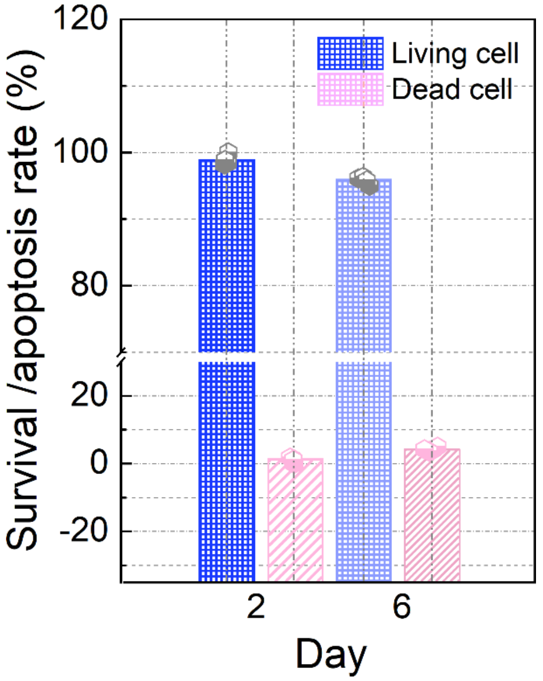

Supplementary Fig. 26. Survival and apoptosis rate statistics of cell cultures on days 2 and 6.

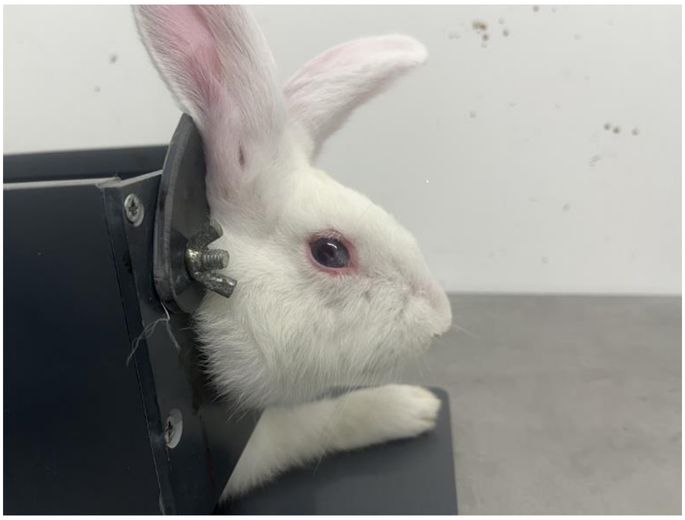

Supplementary Fig. 27. Photographs of rabbit wearing $Ti_3C_2T_x$-SCL.

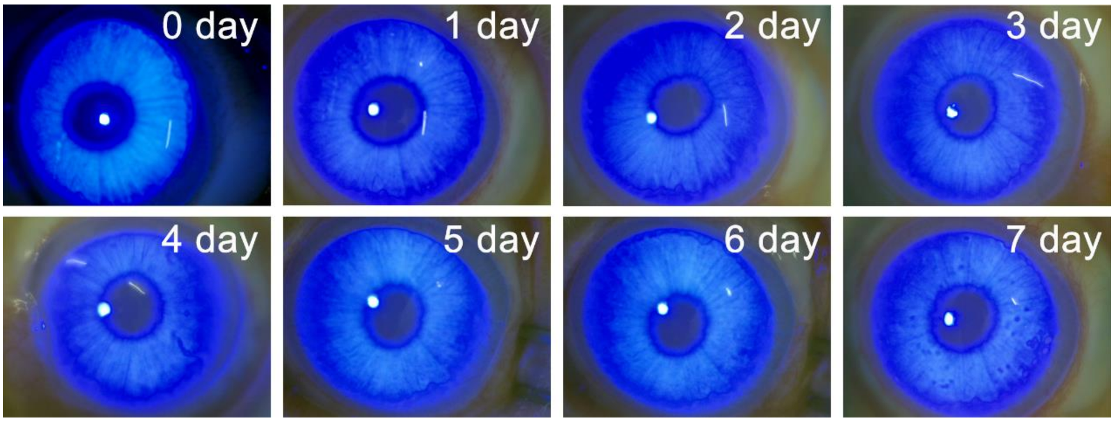

Supplementary Fig. 28. Slit-lamp examination photographs of the rabbit eyes after wearing $Ti_3C_2T_x$-SCL.

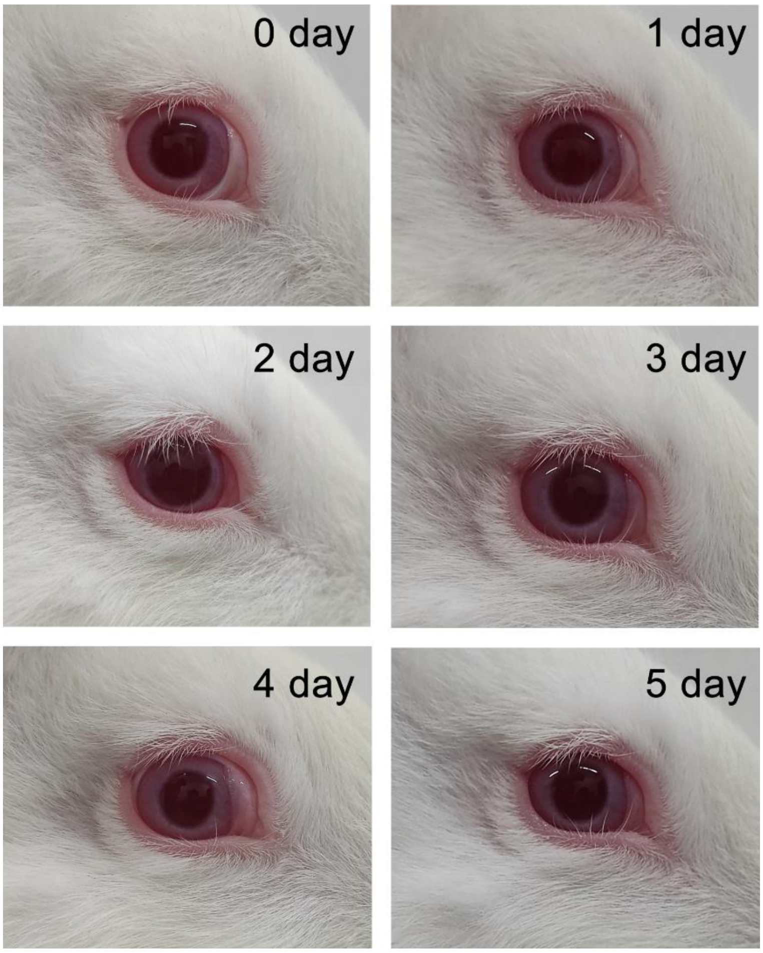

Supplementary Fig. 29. White light photos of the rabbit eyes after wearing $Ti_3C_2T_x$-SCL.

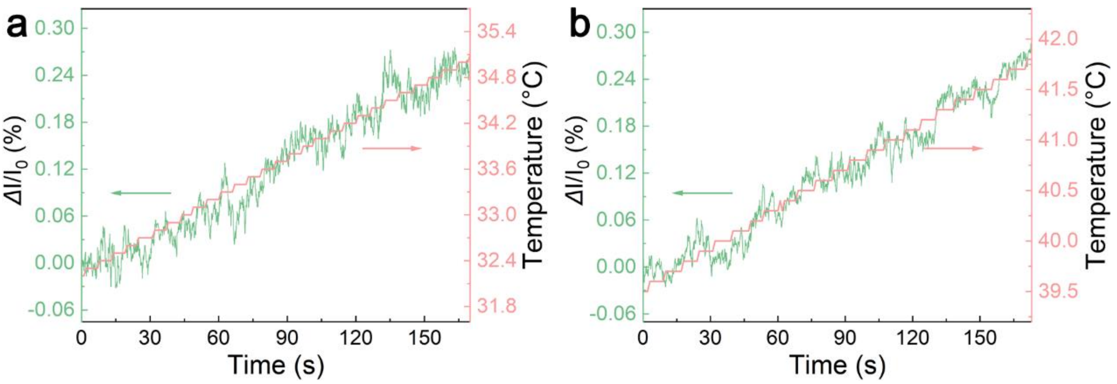

Supplementary Fig. 30. Current dynamic response of temperature sensors during slow temperature rise.

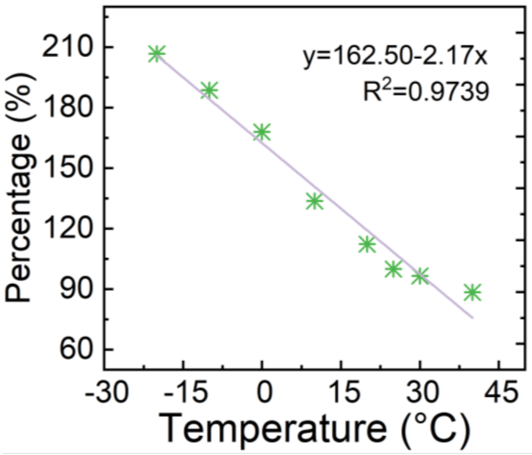

Supplementary Fig. 31. The sensitivity change rate of $Ti_3C_2T_x$-SCL versus temperature over the IOP range of 0-50 mmHg.

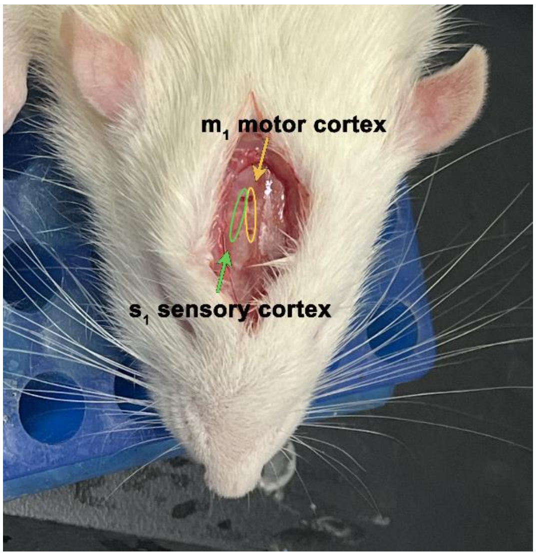

Supplementary Fig. 32. Photograph of rat skull with motor and sensory cortices.

When intraocular pressure (IOP) is elevated, the neuroprosthetic contact lens emits pulsed signals to stimulate the somatosensory cortex. These signals are processed and categorized by the nerve center and transmitted to the motor cortex, which is then synaptically transmits to the sciatic nerve to control the leg twitch.

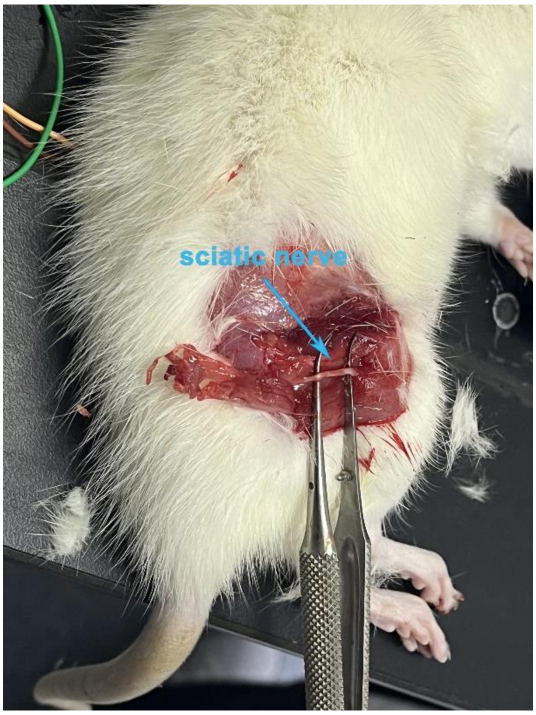

Supplementary Fig. 33. Photograph of the sciatic nerve in a rat.

The neuroprosthetic contact lens can be connected directly to the sciatic nerve and has been used to control leg twitching in rats with abnormal intraocular pressure.

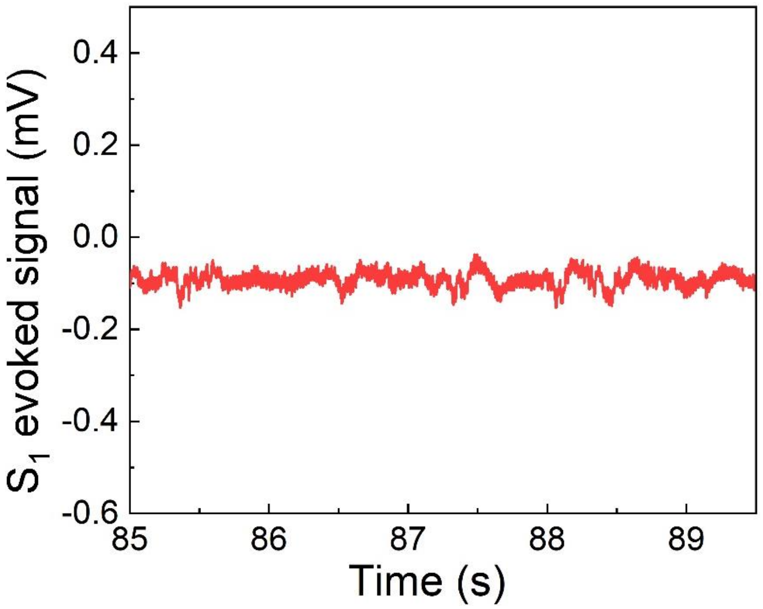

Supplementary Fig. 34. Potential signals from the somatosensory cortex collected during intraocular injection of saline into the rat eye.

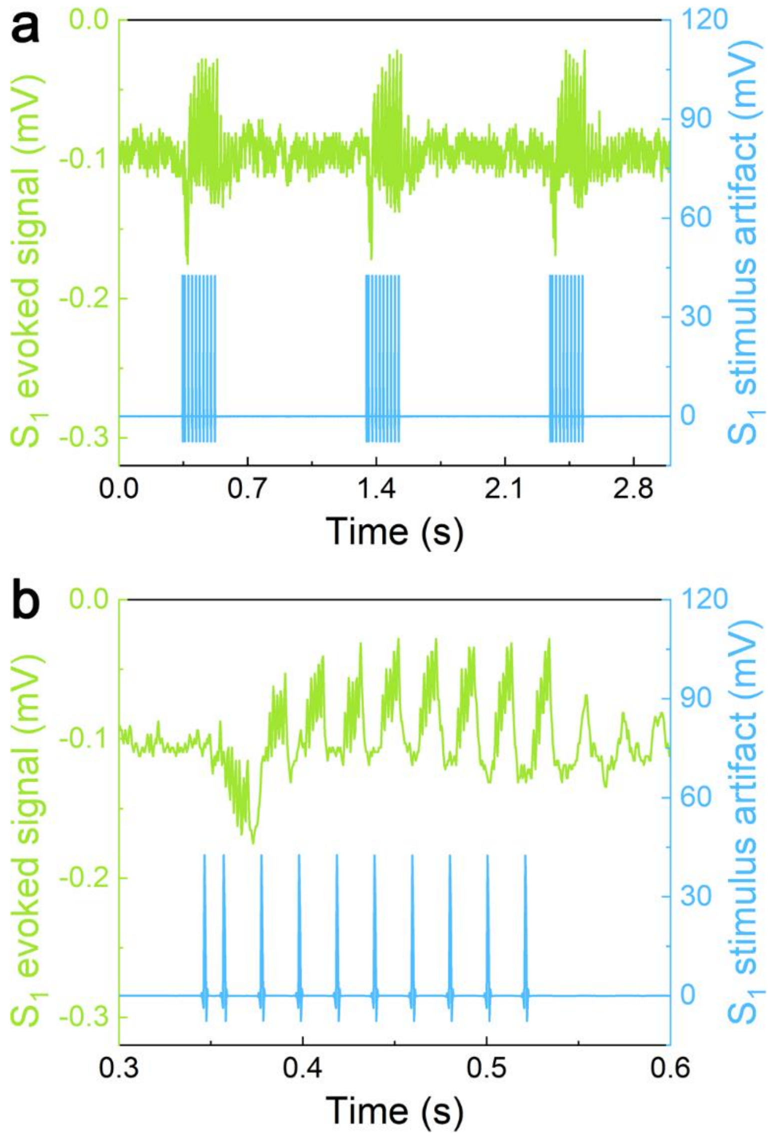

Supplementary Fig. 35. Stimulation of the somatosensory cortex by impulse signals generated by the neuroprosthetic contact lens in response to abnormal intraocular pressure in the rat and the corresponding potential signals collected in the somatosensory cortex, where panel b is a detailed view of a single pulse cluster in panel a.

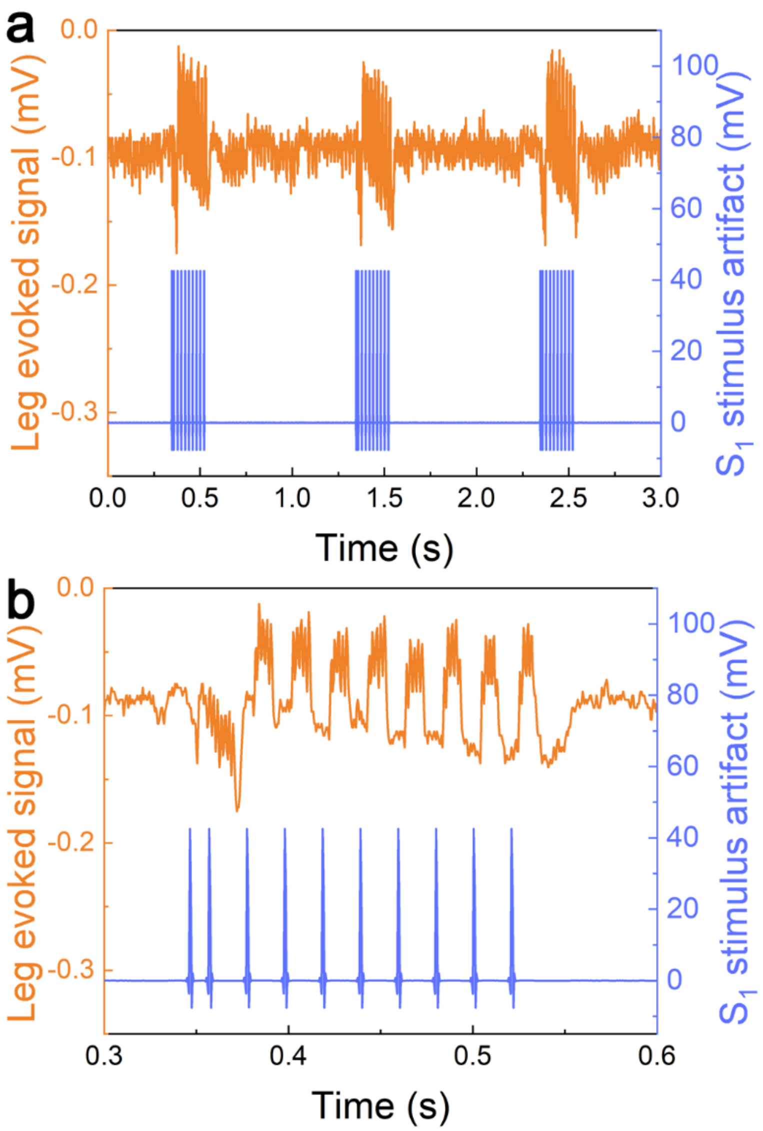

Supplementary Fig. 36. Stimulation of the somatosensory cortex by impulse signals generated by the neuroprosthetic contact lens in response to abnormal intraocular pressure in the rat and the corresponding potential signals collected in the leg, where panel b is a detailed view of a single pulse cluster in panel a.

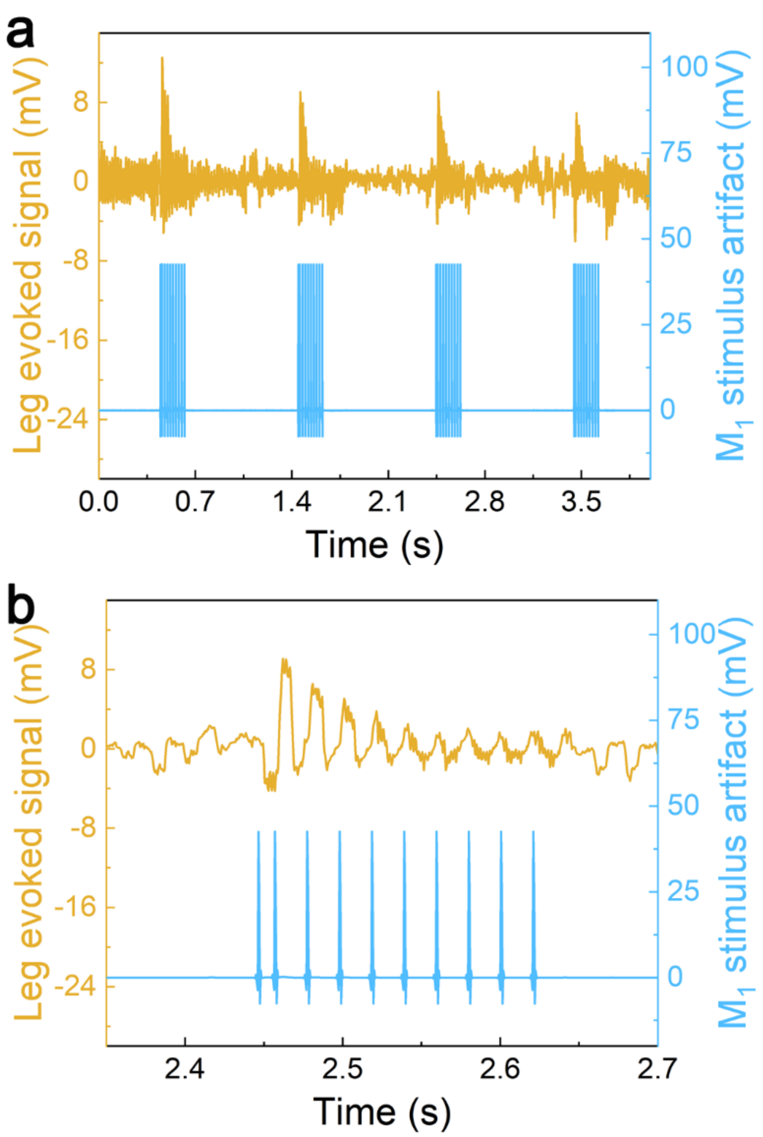

Supplementary Fig. 37. Stimulation of the motor cortex by impulse signals generated by the neuroprosthetic contact lens in response to abnormal intraocular pressure in the rat and the corresponding potential signals collected in the leg, where panel b is a detailed view of a single pulse cluster in panel a.

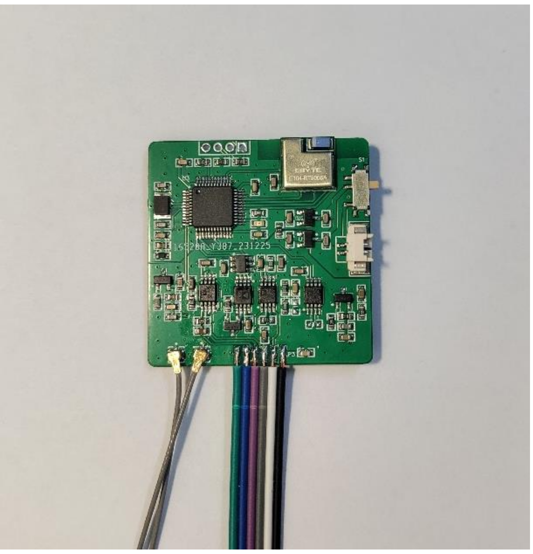

Supplementary Fig. 38. Circuit boards for data acquisition, processing and generation of corresponding stimulus signals.

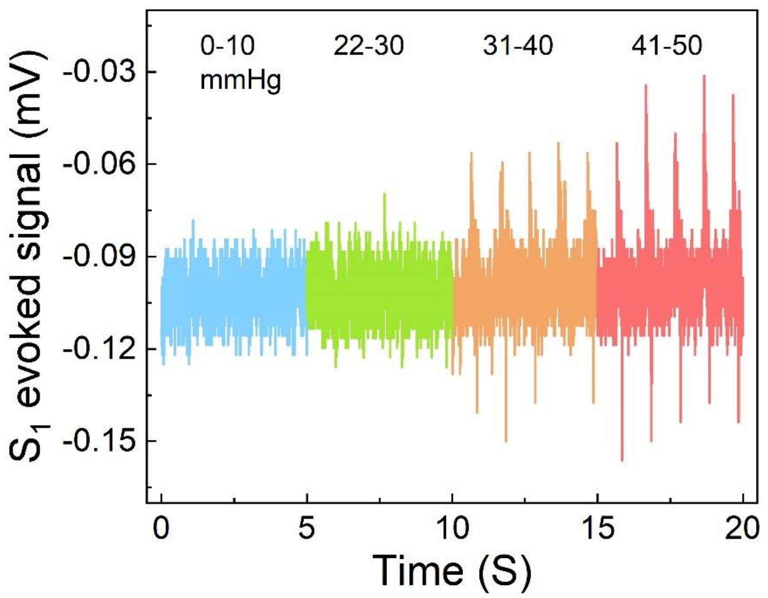

Supplementary Fig. 39. Different levels of the potential signals collected in the somatosensory cortex when the rats were in different IOP ranges.

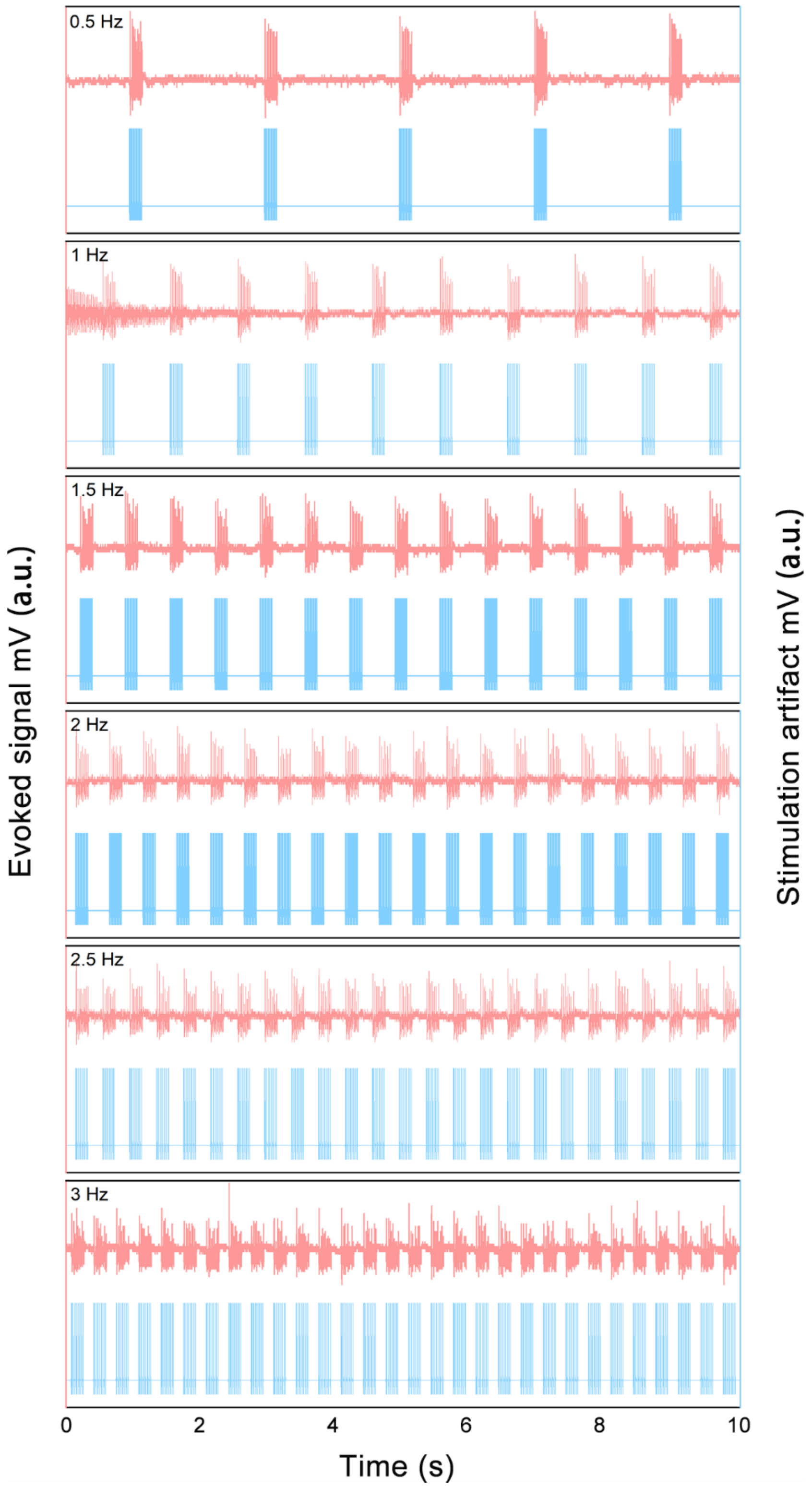

Supplementary Fig. 40. Corresponding signals obtained on the ipsilateral gastrocnemius muscle by stimulating the rat sciatic nerve at different frequencies with a current of 0.1 mA.

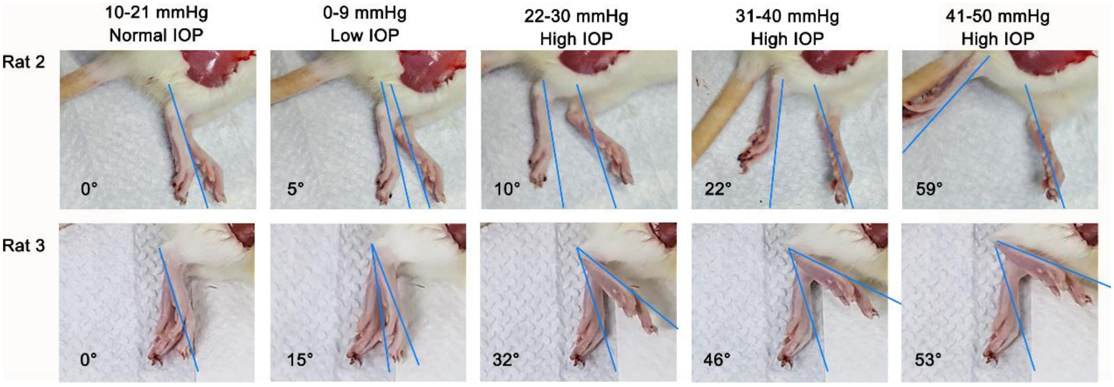

Supplementary Fig. 41. Pictures of the corresponding flexor responses of the rat leg when the intraocular pressure is in different horizontal ranges.

The IOP sensorimotor circuits were constructed on three rats including the one shown in the main text Fig. 5c, which discriminated between normal IOP, high IOP, and low IOP and produced graded motor feedback.

Supplementary Table 1: Weight comparison of $Ti_3C_2T_x$-SCL with commercial transparent and color contact lenses (Unit: g).

Decive	Cooper Optics		Bausch&Lomb		Hydron		$Ti_3C_2T_x$-SCL
	CCL	TCL	CCL	TCL	CCL	TCL	
1	0.0394	0.0357	0.0355	0.0408	0.0366	0.0356	0.0314
2	0.0389	0.0349	0.0357	0.0405	0.0366	0.0343	0.0315
3	0.0394	0.035	0.0362	0.0416	0.0382	0.0348	0.0313
4	0.0389	0.0345	0.0357	0.0408	0.0379	0.0344	0.0314
5	0.0397	0.0351	0.036	0.0412	0.037	0.033	0.0316
6	0.0386	0.0356	0.0354	0.0409	0.0363	0.032	0.0313
7	0.04	0.036	0.0354	0.0408	0.0367	0.0333	0.0314
8	0.0391	0.0355	0.04	0.0408	0.0366	0.0334	0.0316
9	0.0393	0.0363	0.038	0.0412	0.0372	0.035	0.0315
10	0.0386	0.036	0.036	0.041	0.037	0.0332	0.0313
average	0.0392	0.0355	0.0364	0.0410	0.0370	0.0339	0.0314

Supplementary Table 2: Performance comparison with reported articles.

Sensing material	Sensitivity	Measuring range	Reference
platinum-titanium	0.008 mV $mmHg^{-1}$	17-29 mmHg	7
platinum-titanium	0.02 mV $mmHg^{-1}$	10-30 mmHg	8
platinum-titanium	0.109 mV $mmHg^{-1}$	11-30 mmHg	9
platinum-titanium	0.290 mV $mmHg^{-1}$	15-35 mmHg	10
CVD graphene	0.15 mV $mmHg^{-1}$	10-35 mmHg	11
Self-Assembly graphene	1.047 mV $mmHg^{-1}$	10-50 mmHg	12
GO-CNT	0.036 mV $mmHg^{-1}$	9-34 mmHg	13
Graphene/ AgNWs	2.640 MHz $mmHg^{-1}$	0-50 mmHg	14
Cu Capacitive	0.023 MHz $mmHg^{-1}$	5-40 mmHg	15
BL film	1.5 Ω $mmHg^{-1}$	0-52 mmHg	16
Graphene Woven	6.8%	0-15 mmHg	17
**This work- rectilinear**	3.8 mV $mmHg^{-1}$	0-50 mmHg	--
**This work- serpentine**	12.52 mV $mmHg^{-1}$	0-50 mmHg	--

Supplementary Table 3: Standard value, average deviation and accuracy of the $Ti_3C_2T_x$ -SCL at 0.2 mmHg.

Decive	Standard value (mV)	Average variation (mV)	Accuracy (%)
1	2.715	0.026	0.955
2	2.731	0.025	0.899
3	2.749	0.025	0.902
4	2.76	0.024	0.856
5	2.797	0.033	1.173
6	2.821	0.028	0.999
7	2.790	0.022	0.774
8	2.774	0.033	1.189
9	2.746	0.039	1.408
10	2.705	0.030	1.108
11	2.675	0.030	1.111
12	2.675	0.029	1.083
13	2.649	0.028	1.071
14	2.638	0.040	1.517
15	2.583	0.028	1.071
**Average value**	2.721	0.029	1.075

Supplementary Table 4: Maximum measurement error, minimum measurement error and average measurement error at 0.05, 0.1, 0.15, 0.2 and 0.25 mmHg (Unit: mV).

Decive		0.05 mmHg	0.1 mmHg	0.15 mmHg	0.2 mmHg	0.25 mmHg	average
1	max	0.145	0.084	0.069	0.084	0.097	0.096
	min	-0.098	-0.114	-0.064	-0.083	-0.084	-0.089
	average	0.036	0.028	0.028	0.026	0.029	0.029
2	max	0.088	0.073	0.086	0.092	0.096	0.087
	min	-0.079	-0.080	-0.074	-0.090	-0.083	-0.081
	average	0.029	0.028	0.031	0.025	0.029	0.028
3	max	0.123	0.087	0.061	0.076	0.127	0.095
	min	-0.099	-0.132	-0.082	-0.111	-0.121	-0.109
	average	0.034	0.035	0.025	0.025	0.033	0.030
4	max	0.110	0.125	0.111	0.063	0.084	0.098
	min	-0.098	-0.075	-0.103	-0.066	-0.083	-0.085
	average	0.032	0.026	0.028	0.024	0.033	0.028
5	max	0.110	0.086	0.082	0.073	0.12	0.094
	min	-0.114	-0.078	-0.085	-0.087	-0.115	-0.096
	average	0.028	0.031	0.027	0.033	0.036	0.031
6	max	0.085	0.071	0.092	0.059	0.12046	0.086
	min	-0.060	-0.063	-0.087	-0.072	-0.0965	-0.076
	average	0.022	0.028	0.029	0.028	0.03406	0.028
7	max	0.075	0.088	0.078	0.080	0.0694	0.078
	min	-0.068	-0.112	-0.070	-0.046	-0.07126	-0.074
	average	0.025	0.032	0.029	0.022	0.026	0.027
8	max	0.101	0.068	0.080	0.076	0.06679	0.078
	min	-0.103	-0.078	-0.080	-0.081	-0.05957	-0.080
	average	0.038	0.030	0.026	0.029	0.02519	0.030
**average**		0.030	0.030	0.028	0.026	0.031	0.029

Supplementary Table 5: Value-added rate statistics table.

	OD 1	OD 2	OD 3	average value	Subtract blank value	Appreciation Rate
blank group	0.158	0.152	0.151	0.154	--	--
normal group	0.558	0.567	0.563	0.563	0.409	100.00%
Material group –day 1	0.579	0.571	0.569	0.573	0.419	102.53%
Material group -day 2	0.709	0.711	0.729	0.716	0.563	137.57%
Material group -day 3	0.958	0.942	0.956	0.952	0.798	195.19%
Material group -day 4	1.161	1.143	1.155	1.153	0.999	244.34%
Material group -day 5	1.335	1.326	1.334	1.332	1.178	288.02%
Material group -day 6	1.393	1.405	1.418	1.405	1.252	306.03%
Material group -day 7	1.426	1.409	1.421	1.419	1.265	309.29%

Supplementary Table 6: Survival index statistical table of cell in media containing $Ti_3C_2T_x$-SCL within 7 days.

Number	Dead cell count	Viable cell count	Total cell count	Survival rate	Apoptosis rate	Survival index	Apoptotic index
Day 1	0	31	31	100.00%	0.00%	100.00%	0.00%
	0	34	34	100.00%	0.00%		
	0	39	39	100.00%	0.00%		
Day 2	1	62	63	98.41%	1.59%	98.88%	1.12%
	0	64	64	100.00%	0.00%		
	1	55	56	98.21%	1.79%		
Day 3	0	56	56	100.00%	0.00%	99.52%	0.48%
	0	80	80	100.00%	0.00%		
	1	69	70	98.57%	1.43%		
Day 4	2	77	79	97.47%	2.53%	98.71%	1.29%
	0	84	84	100.00%	0.00%		
	1	74	75	98.67%	1.33%		
Day 5	2	72	74	97.30%	2.70%	97.64%	2.36%
	2	97	99	97.98%	2.02%		
	2	83	85	97.65%	2.35%		
Day 6	4	98	102	96.08%	3.92%	95.80%	4.20%
	5	95	100	95.00%	5.00%		
	4	105	109	96.33%	3.67%		
Day 7	12	142	154	92.21%	7.79%	94.69%	5.31%
	3	128	131	97.71%	2.29%		
	8	129	137	94.16%	5.84%		
Control group	0	20	20	100.00%	0.00%	100.00%	0.00%
	0	21	21	100.00%	0.00%		
	0	28	28	100.00%	0.00%		

References

1 Liu, W., Li, L. & Shen, G. A $Ti_3C_2T_x$ MXene cathode and redox-active electrolyte based flexible Zn-ion microsupercapacitor for integrated pressure sensing application. *Nanoscale* **15**, 2624–2632 (2023).

2 Sarycheva, A. & Gogotsi, Y. Raman spectroscopy analysis of the structure and surface chemistry of $Ti_3C_2T_x$ MXene. *Chem. Mater.* **32**, 3480–3488 (2020).

3 Liu, W., Li, L., Hu, C., Chen, D. & Shen, G. Intercalation of small organic molecules into $Ti_3C_2T_x$ MXene Cathodes for flexible high-volume-capacitance Zn-ion microsupercapacitor. *Adv. Mater. Technol.* **7**, 2200158 (2022).

4 Han, M., et al. Ti_3C_2 MXenes with Modified Surface for High-Performance Electromagnetic Absorption and Shielding in the X-Band. *ACS Appl. Mater. Interfaces* **8,** 21011–21019 (2016).

5 Shao, B., et al. Synthesis of 2D/2D CoAl-LDHs/$Ti_3C_2T_x$ Schottky-junction with enhanced interfacial charge transfer and visible-light photocatalytic performance. *Appl. Catal. B* **286,** 119867 (2021).

6 Hu, C., Du, Z., Wei, Z., Li, L. & Shen, G. Functionalized $Ti_3C_2T_x$ MXene with layer-dependent band gap for flexible NIR photodetectors. *Appl. Phys. Rev.* **10**, 021402 (2023).

7 Leonardi, M., Leuenberger, P., Bertrand, D., Bertsch, A. & Renaud, P. First steps toward noninvasive intraocular pressure monitoring with a sensing contact lens. *Invest. Ophthalmol. Vis. Sci.* **45**, 3113 (2004).

8 Xu, J. *et al.* Highly transparent and sensitive graphene sensors for continuous and non-invasive intraocular pressure monitoring. *ACS Appl. Mater. Inter.* **12**, 18375–18384 (2020).

9 Leonardi, M., Pitchon, E. M., Bertsch, A., Renaud, P. & Mermoud, A. Wireless contact lens sensor for intraocular pressure monitoring: assessment on enucleated pig eyes. *Acta Ophthalmol.* **87**, 433–437 (2009).

10 Dou, Z. *et al.* Wearable contact lens sensor for non-invasive continuous monitoring of intraocular pressure. *Micromachines* **12**, 108 (2021).

11 Pang, Y. *et al.* A contact lens promising for non-invasive continuous intraocular pressure monitoring. *RSC Adv.* **9**, 5076–5082 (2019).

12 Liu, Z. *et al.* An ultrasensitive contact lens sensor based on self-assembly graphene for continuous intraocular pressure monitoring. *Adv. Funct. Mater.* **31**, 2010991 (2021).

13 Zhang, Y. *et al.* High resolution non-invasive intraocular pressure monitoring by use of graphene woven fabrics on contact lens. *Microsyst. Nanoeng.* **5**, 39 (2019).

14 Fan, Y. *et al.* A wearable contact lens sensor for noninvasive in-situ monitoring of intraocular pressure. *Nanotechnol.* **32**, 095106 (2021).

15 Chen, G.-Z., Chan, I.-S. & Lam, D. C. C. Capacitive contact lens sensor for continuous non-invasive intraocular pressure monitoring. *Sensor. Actuat. A-phys.* **203**, 112–118 (2013).

16 Kim, J. *et al.* Wearable smart sensor systems integrated on soft contact lenses for wireless ocular diagnostics. *Nat. Commun.* **8**, 14997 (2017).

17 Laukhin, V. *et al.* Non-invasive intraocular pressure monitoring with a contact lens engineered with a nanostructured polymeric sensing film. *Sensor. Actuat. A-phys.* **170**, 36–43 (2011).
