# Supplementary material for: Neuroprosthetic contact lens enabled sensorimotor system for point-of-care monitoring and feedback of intraocular pressure
Source: Nat Commun. 2024 Jul 5;15:5635. doi: 10.1038/s41467-024-49907-5 (PMC11224243; doi:10.1038/s41467-024-49907-5)
Supplement: Supplementary file 3 — Description of Additional Supplementary Files [file 41467_2024_49907_MOESM3_ESM.pdf]

Description of Additional Supplementary Files

File Name: Supplementary Movie 1
Description: Comfort validation of neuroprosthetic contact lens in rabbit eyes.

File Name: Supplementary Movie 2
Description: Infrared thermal imaging process cooling from 40 ºC to -20 ºC.

File Name: Supplementary Movie 3
Description: Motor cortex-controlled feedback.

File Name: Supplementary Movie 4
Description: IOP sensorimotor loop reconstruction in rat with neuroprosthetic contact lens.
